# Transcriptome profile of pecan scab resistant and susceptible trees from a pecan provenance collection

**DOI:** 10.1186/s12864-024-10010-0

**Published:** 2024-02-15

**Authors:** Jordan Brungardt, Yanina Alarcon, Jason Shiller, Carolyn Young, Maria J. Monteros, Jennifer J. Randall, Clive H. Bock

**Affiliations:** 1 USDA-ARS, Byron, GA USA; 2 Noble Research Institute, Ardmore, OK USA; 3 University of Texas Southwestern, Dallas, TX USA; 4 The New Zealand Institute for Plant and Food Research, Auckland, New Zealand; 5 Entomology and Plant Pathology, NC State University, Raleigh, NC USA; 6 Bayer Crop Science, Chesterfield, MO USA; 7 New Mexico State University, Las Cruces, NM USA

**Keywords:** Pecan, Scab, RNA-seq, DEG, Fungal resistance, Venturia, Carya, Provenance

## Abstract

Pecan scab is a devastating disease that causes damage to pecan (*Carya illinoinensis* (Wangenh.) K. Koch) fruit and leaves. The disease is caused by the fungus *Venturia effusa* (G. Winter) and the main management practice for controlling the disease is by application of fungicides at 2-to-3-week intervals throughout the growing season. Besides disease-related yield loss, application of fungicides can result in considerable cost and increases the likelihood of fungicide resistance developing in the pathogen. Resistant cultivars are available for pecan growers; although, in several cases resistance has been overcome as the pathogen adapts to infect resistant hosts. Despite the importance of host resistance in scab management, there is little information regarding the molecular basis of genetic resistance to pecan scab.

The purpose of this study was to elucidate mechanisms of natural pecan scab resistance by analyzing transcripts that are differentially expressed in pecan leaf samples from scab resistant and susceptible trees. The leaf samples were collected from trees in a provenance collection orchard that represents the natural range of pecan in the US and Mexico. Trees in the orchard have been exposed to natural scab infections since planting in 1989, and scab ratings were collected over three seasons. Based on this data, ten susceptible trees and ten resistant trees were selected for analysis. RNA-seq data was collected and analyzed for diseased and non-diseased parts of susceptible trees as well as for resistant trees. A total of 313 genes were found to be differentially expressed when comparing resistant and susceptible trees without disease. For susceptible samples showing scab symptoms, 1,454 genes were identified as differentially expressed compared to non-diseased susceptible samples. Many genes involved in pathogen recognition, defense responses, and signal transduction were up-regulated in diseased samples of susceptible trees, whereas differentially expressed genes in pecan scab resistant samples were generally down-regulated compared to non-diseased susceptible samples.

Our results provide the first account of candidate genes involved in resistance/susceptibility to pecan scab under natural conditions in a pecan orchard. This information can be used to aid pecan breeding programs and development of biotechnology-based approaches for generating pecan cultivars with more durable scab resistance.

**Supplementary Information:**

The online version contains supplementary material available at 10.1186/s12864-024-10010-0.

## Introduction

Pecan (*Carya illinoinensis* (Wangenh.) K. Koch; *C. illinoinensis*) is native to North America, and is one of the most important commercial nut trees in the United States. In a particularly good year, 2017, pecan production for the US was estimated to be worth around $700 million [1]. The native range of pecan extends from the Mississippi River Watershed in the US to southern Mexico [2]. A provenance collection including germplasm from throughout the native range was established in the late 1980s, and is planted at the USDA-Agriculture Research Service (ARS) – Southeastern Fruit and Tree Nut Research Station (SEFTNRS) at Byron, GA [3]. The provenance collection is a unique genetic resource that can be used to characterize multiple horticulturally important traits including scab resistance [4, 5].

Pecan scab (caused by the plant pathogenic fungus *Venturia effusa* (G. Winter)) is a major constraint to pecan production in the southeastern US. Pecan scab reduces nut quality and yield [6]. This ascomycete pathogen infects leaves, nuts and twigs when weather conditions are conducive, particularly during periods of rainfall, warmth and high humidity [7]. Leaves are susceptible to the pathogen during their expansion [7], whereas nuts are susceptible throughout their development and maturation stages. Until recently *V. effusa* was believed to reproduce solely through the production of asexual conidia, which are considered the primary source of inoculum [8]. However, the recent discovery of a sexual stage of *V. effusa* has been observed through *in vitro* mating of diverse isolates by Charlton et al. [9]. Identification of the sexual stage is consistent with studies that have shown populations of *V. effusa* to be genetically diverse, heterothallic, with both mating types present in equilibrium [10, 11]. Thus, the sexual stage likely plays a role yet to be elucidated in the lifecycle and epidemic development of pecan scab. Although pecan scab may be managed through tree hedging, tree thinning, and use of fungicides, these increase on farm expenses and reduce profitability. The annual cost of fungicide application combined with the inevitable yield loss due to scab is substantial [12]. Moreover, *V. effusa* has developed resistance to several classes of fungicides, which can present challenges to effective management of the disease in production orchards (Reviewed in: [13]). Thus, understanding the mechanisms of resistance to scab that exist in a diverse collection of pecan is highly desirable to guide breeding programs and develop biotechnology approaches for durable scab resistance.

Plant responses to biotic stress are the result of complex processes comprising of pathogen detection and signal transduction mechanisms (Reviewed in: [14, 15]). The first line of defense plants use for sensing an attacking pathogen is to detect molecules that are common to different types of microbes. These molecules are commonly referred to as pathogen/microbe associated molecular patterns (PAMP/MAMP) and are detected by pathogen recognition receptors (PRR) and kinases with transmembrane domains. This type of immunity is known as pattern triggered immunity (PTI). Pathogens are able to overcome PTI by disrupting the immune response with molecules known as effectors. In return, plants have evolved mechanisms for detecting pathogen effectors in what is known as effector triggered immunity (ETI). Among an array of different types of effector receptors, nucleotide binding leucine-rich repeat domain containing proteins (NB-LRR) are the main source of ETI resistance genes [16]. The ETI resistance genes are highly specific to each plant pathosystem. Short range signal transduction mechanisms involve different chemical pathways typically involving mitogen-activated protein (MAP) kinases [17]. Ultimately, pathogen recognition and signal transduction drive the expression of pathogenesis related genes and defense responses. The epitome of plant defense response is characterized by a hypersensitive response (HR), in which reactive-oxygen species (ROS) are produced followed by programmed cell death. Also, production of chitinases, defensins, thaumatins, and secondary metabolites are additional methods plants use to defend against pathogens [18]. Due to the intricacies present in any particular plant pathosystem, a thorough genetic investigation is required to elucidate mechanisms of resistance. Durable resistance for plants often requires multiple resistance genes, as a compatible interaction between effector and R-gene exhibits a strong negative selection on the pathogen and subsequent effector. Stacking multiple resistance genes has been reported in rice for resistance to bacterial diseases [19], and resistance to fungal disease in apple [20] and sunflower [21]. The relatively long time required for pecan trees to reach maturity and start setting fruit would make stacking R-genes a lengthy endeavor.

Pecan is a perennial, outcrossing plant species with cultivars that are propagated through grafting. Rootstocks are known to be a source of variation for different traits in several fruit trees including apple [22] and citrus [23]. One approach for identifying genes involved with pecan scab resistance/susceptibility, without the added potential of variation from rootstock, is to obtain transcriptome profiles from seed-grown trees like those present in the pecan provenance collection. Such a strategy is able to explore pecan responses to a naturally occurring scab population comprising a diverse population of *V. effusa* pathovars [24]. This in conjunction with the genetic diversity of the provenance collection should provide the framework to identify multiple resistant/susceptibility mechanisms in the pecan/*V. effusa* pathosystem. Pecan derived from northern germplasm, as opposed to Mexico and Texas germplasm, is generally more resistant to scab. Transcriptome profiling using RNA-Sequencing (RNA-seq) captures the transcriptome profile of an individual at a given time and environmental condition. Comparing transcript profiles between multiple conditions allows one to identify differentially expressed genes (DEG). Differential expression studies have been previously reported for fruit and nut tree species for biotic stresses under field conditions [25–27]. Numerous differential expression studies have been done on *C. illinoinensis* in the past, most of which address horticultural questions [28–31]. Only recently has this method been used to examine biotic stress in pecan. Chen et al. examined transcript profiles of three different pecan cultivars with varying levels of resistance to the ascomycete *Pestalotiopsis microspora* [32]. In regards to *V. effusa*, Lovell *et al*. used RNAseq to find DEGs in pecan after inoculation with *V. effusa* under controlled conditions; however, only a single cultivar-pathotype interaction was studied and there was no evaluation of resistant tree genotypes [33].

For the present study, a subset of pecan trees from the provenance collection that were consistently identified as scab resistant or susceptible during three years of disease assessment in the orchard were used for differential expression analysis [4, 5]. In addition, diseased and disease-free leaves from susceptible trees were collected and analyzed. RNA-Seq of leaf samples enabled transcriptome profiling of the trees for the identification of genes likely involved in scab resistance/susceptibility. Gene ontology (GO) enrichment analysis was used for the identification of functional categories of DEGs and to explore their potential role in the pecan scab pathosystem.

## Materials and methods

### Plant materials, scab assessments and RNA extraction

Scab evaluations and leaf samples were taken from trees in the *C. illinoinensis* provenance collection located at the USDA-ARS SEFTNRS station in Byron, GA. Scab severity was evaluated on leaves during the growing seasons of 2013 and 2014 by Bock et al. [5], as well as on leaves and fruit in 2018 [4]. Based on the scab assessments, ten consistently scab resistant and ten consistently susceptible trees were selected for the current study (Table 1).

**Table 1:** Pecan scab severity in 2013 and 2014 on leaves (mean severity) and in 2018 on leaves and nuts (most severe and overall tree severity) for provenance trees at Byron, GA

**Provenance-family (mother tree)**^**a**^	**Tree ID number**	**Scab severity**						**Overall scab response**
		**2013 (foliar % severity)**	**2014 (foliar % severity)**	**2018 (most severe foliar % severity)**	**2018 (overall tree foliar % severity)**	**2018 (most severe nut % severity)**	**2018 (overall tree nut % severity)**	
TX2-2	11	12.6	1.2	10	10	60	30	Susceptible
MX4-2	18	5.2	7.3	80	5	70	60	Susceptible
TX3-4	42	3.1	6.6	50	10	90	60	Susceptible
MX1-1	46	3.4	1.4	50	2	70	40	Susceptible
TX2-2	229	3.9	1.3	70	10	100	90	Susceptible
TX5-4	266	2.4	2.2	20	1	70	20	Susceptible
TX2-5	283	2.1	5.4	70	10	90	30	Susceptible
TX3-4	505	2.2	3.5	50	5	100	10	Susceptible
TX5-5	491	1.2	1.3	30	2	60	60	Susceptible
TX3-3	571	2.4	4.5	20	2	100	90	Susceptible
KS2-3	16	0	0	0	0	0	0	Resistant
TX4-2	80	0	0	0	0	0	0	Resistant
MO3-2	116	0	0	0	0	0	0	Resistant
MO1-5	155	0	0	0	0	0	0	Resistant
TN1-1	163	0	0	0	0	0	0	Resistant
MX5-2	268	0	0	0	0	0	0	Resistant
IL1-2	423	0	0	0	0	0	0	Resistant
TN1-1	497	0	0	0	0	0	0	Resistant
KY1-3	560	0	0	0	0	0	0	Resistant
IL1-5	541	0	0	0	0	0	0	Resistant

^a^Provenances are indicated as IL1 (Jersey Co., Illinois), KS2 (Cherokee Co., Kansas), KY1 (Webster Co., Kentucky), MO1 (Livingston Co., Missouri), MO3 (Vernon Co., Missouri), MX1 (Santa Catarina, San Luis Potosi, Mexico), MX4 (Ixmiquilpan, Mexico), MX5 (Jaumave, Mexico), TN1 (Lake Co., Tennessee), TX2 (Zavala Co., Texas), TX3 (Kinney Co., Texas), TX4 (Tom Green Co., Texas), TX5 (Val Verde Co., Texas) (Ref. 3)

For susceptible trees, two sets of samples were taken from each tree, leaves with scab symptoms (S-D = susceptible diseased) and leaves with no scab symptoms (S-ND = susceptible no disease). Only one set of samples (*R* = resistant) was taken from resistant trees as no disease was evident. Leaf tissue (five leaflets per tree) was collected at one-third total leaf expansion from the twenty trees early in the scab season on April 30th of 2019, when symptoms of scab were easily detected and not confounded by other diseases or pest damage that can occur late in the season. Scissors, sterilized between leaflet samples with 70% ethanol, were used to excise leaflets. Powder free gloves (a new pair for each sample, sterilized with ethanol) were worn during sampling and the samples were placed in RNAase free tubes containing Invitrogen RNAlater^TM^ (ThermoFisher Scientific; MA, US) to store the leaf samples for further processing after sampling. At the time of RNA extraction, RNAlater^TM^ was removed from the samples, the leaf tissue was ground in liquid nitrogen, and RNA extracted using the Spectrum™ Plant Total RNA Kit (Sigma Aldrich; MO, US). A Bioanalyzer 2100 (Agilent Inc.; CA, US) was used to ensure samples had an RNA integrity number greater than seven.

### Pecan RNA-Seq library preparation and differential gene expression determination

RNA-seq libraries were constructed by first polyA purifying 100 ng total RNA with TruSeq® Stranded mRNA Sample Prep kits (Illumina, CA, US) following standard protocols. Sixty individual libraries (two technical replicates of ten S-ND, S-D, and R samples) were uniquely indexed using TruSeq RNA Single Indexes (Illumina, CA, US). The pooled libraries were sequenced using an Illumina NovaSeq 6000, paired-end 150 bp at Novogene Inc. (CA, US). Raw reads can be accessed through the National Center for Biotechnology’s Sequence Read Archive (NCBI-SRA) as Bioproject PRJNA992068. The resulting reads were quality trimmed with Trimmomatic 0.38 using the following parameters [34].

ILLUMINACLIP:TruSeq3-PE.fa:2:30:10:3:TRUE HEADCROP:17 LEADING:3 TRAILING:3 SLIDINGWINDOW:4:24 MINLEN:100

The quality of sequence reads was verified with Fastqc 0.11.9 by comparing reads before and after trimming. One resistant replicate was not used for analysis due to low read quality. Reads were aligned to the Phytozome V13, 573 *C. illinoinensis* 87MX3-2.11 annotation version 1.1 transcripts and primary coding sequence (CDS) transcripts [35] separately with Bowtie2 v3.4 using default parameters [36]. Sequence alignment/map files were converted to binary alignment/map files with SAMtools 1.10 [37]. Transcript abundance was then quantified using Salmon 0.10.1, correcting for GC bias [38]. Quantified read information was then imported into R [39] for differential analysis with “tximport” using default parameters [40]. Differential analysis was done with DEseq2 [41] using the “ashr” log fold-change shrinkage estimator method [42]. Comparisons made include S-ND leaves vs. R leaves as well as S-ND leaves vs. S-D leaves. Genes that had a Benjamini & Hochberg adjusted *p*-value less than 0.05 and an absolute log base two-fold-change (LFC) of one and a half were considered to be differentially expressed.

### *Venturia effusa* reads in pecan samples

To estimate the relative level of *V. effusa* infection in each pecan sample, previously trimmed sample reads were mapped to the *V. effusa* FRT5LL7-albino genome (Genbank:GCA_007735645.1; Ref. [43]) using Bowtie2 v3.4. Information for percent reads mapped to the *V. effusa* genome and those previously mapped to *C. illinoinensis* primary CDS transcriptome were retrieved from Bowtie2 log files. Analysis of variance and Tukey’s honestly significant difference (HSD) post-hoc tests were performed with the R base statistics package. Due to high sequence similarity of several *C. illinoinensis* and *V. effusa* orthologs, levels of *V. effusa* in samples were also checked with reads mapped to the *V. effusa* high expressing, non-orthologous elongation factor EF-1 alpha transcript ( [43]; Protein ID QDS71019.1).

### Differential expression post hoc analysis

Subsequent analyses were done with data generated from alignments to the primary CDS transcript information. Genes that were differentially expressed in S-ND vs. R and S-ND vs. S-D analyses were visualized with the R package VennDiagram [44]. Annotation information from the Phytozome V13, 573 *C. illinoinensis* 87MX3-2.11 genome version 1.1 (*C. illinoinensis* 573 genome; Ref. 35) was used for enrichment analysis of DEGs with Pathview (https://pathview.uncc.edu/; Accessed Oct. 2022; [45]), Mapman (Data not shown; [46]), and GOrilla (http://cbl-gorilla.cs.technion.ac.il/; Accessed Oct. 2022; [47]). Gene ontology (GO) enrichment was done by identifying DEGs with *Arabidopsis thaliana* (*A. thaliana*) ortholog information as well as GO annotation information from the *C. illinoinensis* 573 assembly annotation information. The resulting information was passed into the online GOrilla analysis tool. Unranked gene lists were used with a *p*-value threshold of 10^-3^, evaluating all ontology types. For Pathview analysis, gene set analysis (GSA) was done by first filtering Salmon normalized counts for DEGs with *A. thaliana* ortholog information. Pathview utilizes Kyoto Encyclopedia of Genes and Genomes (KEGG) pathways to visualize relationships between elements of specific metabolic pathways [48]. Plant pathways of particular interest to interactions with pathogens (KEGG pathways 00999, 04016, 04075, and 4626) were evaluated as well as using the automatic pathway selection feature of Pathview. R-genes were predicted from the *C. illinoinensis* 573 genome with DRAGO2-API (DRAGO; [49]) and NLR-annotation v2.1 [50]. Transcription factors were predicted with DeepTFactor [51] and Plant Transcription Factor Database v 5.0 (PlantTFDB; [52]) using default settings.

To verify results generated from the next generation sequencing (NGS) information, expression of several DEGs was checked with quantitative PCR (qPCR) from one-third expanded leaves collected on May 19^th^, 2023. Tissue was collected from four random trees of the previously identified S-ND and R groups. Tissue was placed into 2 mL microfuge tubes, which were placed directly into liquid nitrogen. Samples were maintained at -80°C until further processing. RNA was extracted using the lignified/polyphenol-rich protocol of the GeneJET Plant RNA purification Mini Kit (ThermoFisher Scientific). Quality of RNA was checked with a Nanodrop spectrophotometer to ensure 260 280^-1^ and 260 230^-1^ ratios > 1.8 and integrity of RNA was checked visually via gel-electrophoresis. Potential genomic DNA (gDNA) was removed from samples by treating 1 µg of sample with RNase-free DNase I following manufacturer’s recommendations (ThermoFisher Scientific) with the addition of 0.5µL RNasin Ribonuclease Inhibitor (Promega) per reaction. First strand complementary DNA (cDNA) was generated using Avian Myeloblastosis Virus reverse transcriptase and oligo(dT) primers (Promega). Presence/absence of gDNA and cDNA was confirmed using PCR with intron spanning primers (Additional file 1; tab 2 ‘Primers’). Each sample/gene-target combination of interest was evaluated on a Bio-Rad CFX Opus 96 Real-Time PCR system using three technical replicates. qPCR reactions used PowerUp^TM^ SYBR^TM^ Green master mix (Applied Biosystems; MA, US) and 0.35 µL undiluted cDNA reaction as a template for each quantitative reaction. Primer information used in qPCR reactions is provided (Additional file 1; tab 2 ‘Primers’). Polyadenylate-binding protein (Caril.16G041200) was chosen as a reference gene due to its small coefficient of variation in expression derived from RNAseq data generated for this study.

## Results

### RNAseq, mapping, and differential gene expression identification

**Fig. 1:**
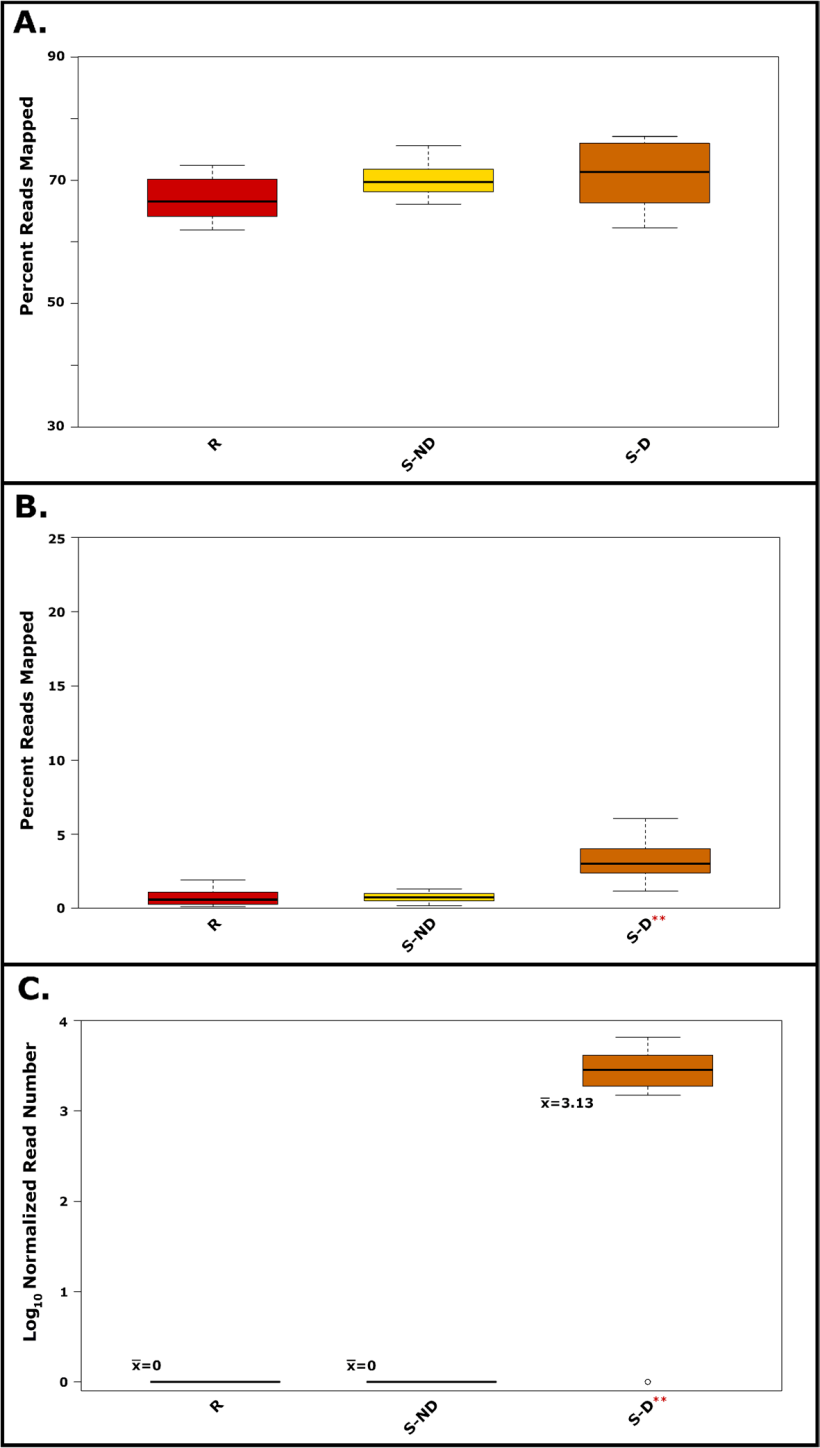
Boxplots of RNAseq data mapped to either the *Carya illinoinensis* transcriptome (**A**) or *Venturia effusa* genome (**B**). RNAseq data was generated from susceptible non-diseased leaves (S-ND), diseased susceptible leaves (S-D), and resistant leaves (R). The double asterisk (**) indicates a Tukey’s HSD *p*-value < 0.05 for a given treatment. Reads mapped to the high expressing, non-orthologous *Venturia effusa* elongation factor one-alpha are shown (**C**). Whiskers represent the minimum of either 1.5 times the interquartile range or the extreme of the sample range. Open circles indicate outliers

RNA sequencing resulted in more than 2.55 billion reads being referenced to sample barcodes. Samples had a mean paired-read count of 43.28 million (s.d. 10.47 mil.). After trimming, approximately 25% of raw reads were removed from each sample. Mapping of RNAseq data was performed separately on two *C. illinoinensis* transcript reference assemblies and a *V. effusa* genome. As expected, reads mapped to a much lesser extent to the fungal genome compared to that of the pecan genome assembly (Fig. 1A & B).

**Fig. 2:**
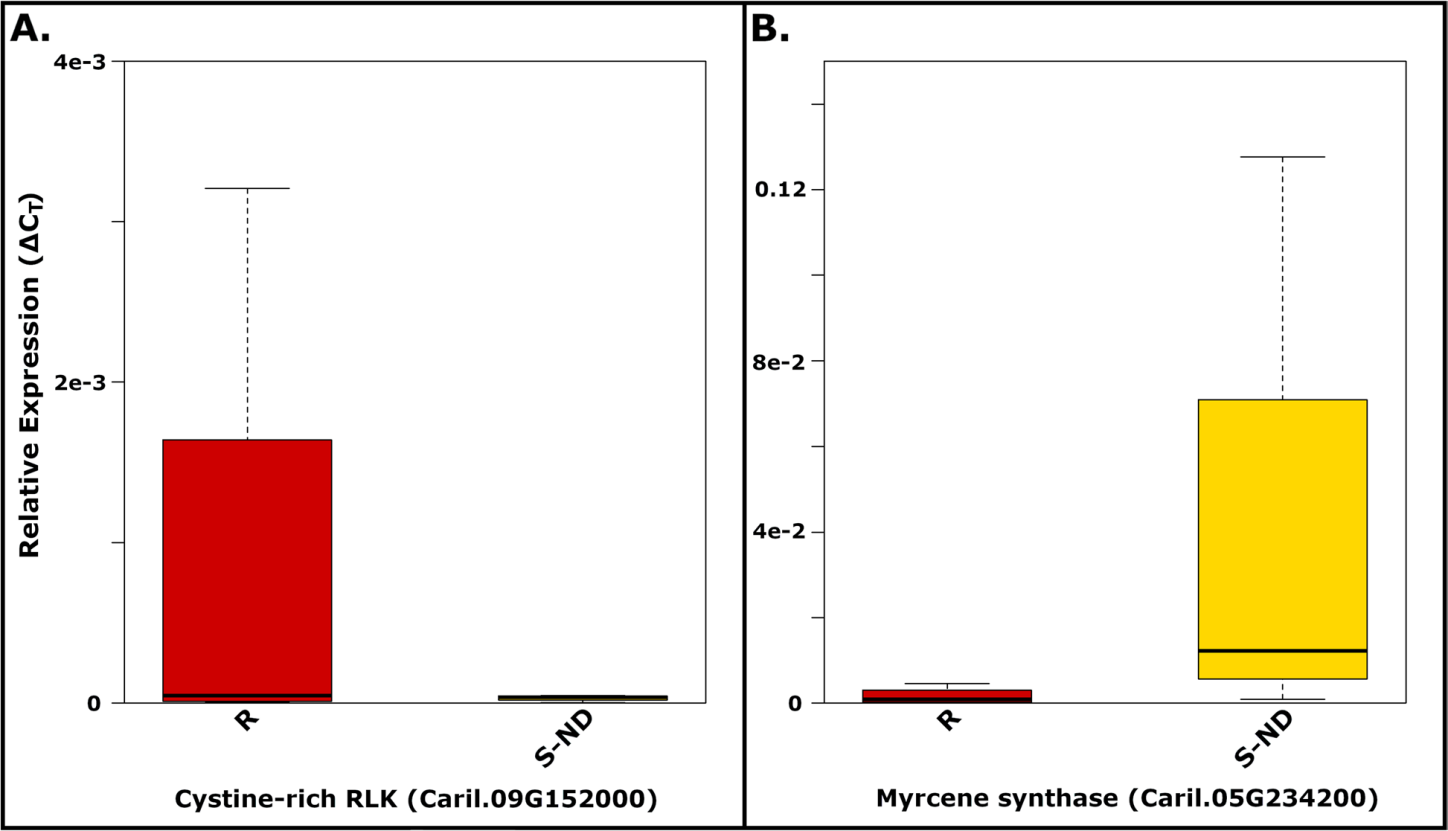
Quantitative PCR showing relative expression of two genes in pecan leaves collected in 2023 (*n*=4) using polyadenylate-binding protein (Caril.16G041200) as a reference gene. Expression patterns were similar to DEGs identified in RNAseq data from samples collected in 2019, showing up-regulation of a cystine-rich receptor-like kinase (Caril.09G152000, **A**) in resistant leaves (R) compared to susceptible non-diseased leaves (S-ND), while myrcene synthase (Caril.05G234200, **B**) was down-regulated in resistant leaves (R)

In addition, the percentage of *V. effusa* reads per sample was much greater for susceptible diseased leaves (S-D; $\overline{x }$=1.72%) compared to non-diseased susceptible (S-ND; $\overline{x }$=0.76%) and resistant leaves (R; $\overline{x }$=0.79%) (Tukey’s HSD *p*-value < 0.05). In fact, no reads mapped to the non-orthologous *V. effusa* elongation factor alpha gene in R and S-ND samples (Fig. 1C). For the pecan assemblies, the average number of reads mapped to the primary CDS assembly was 69.3%, whereas the average number of reads mapped to the isoform containing assembly was 87.3%. Despite this discrepancy, the composition of resulting DEGs between the two assemblies was very similar (data not shown); therefore, the following analyses were performed on DEGs derived from the primary CDS assembly only. Mapping and DEG analysis against the Phytozome V13 *Carya illinoinensis* 588 cv. Pawnee v1.1 (susceptible to pecan scab) primary transcriptome also gave similar results (Ref. 33; data not shown). In addition, the expression pattern of several DEGs was similar in trees sampled at a later time when using qPCR (Fig. 2).

**Fig. 3:**
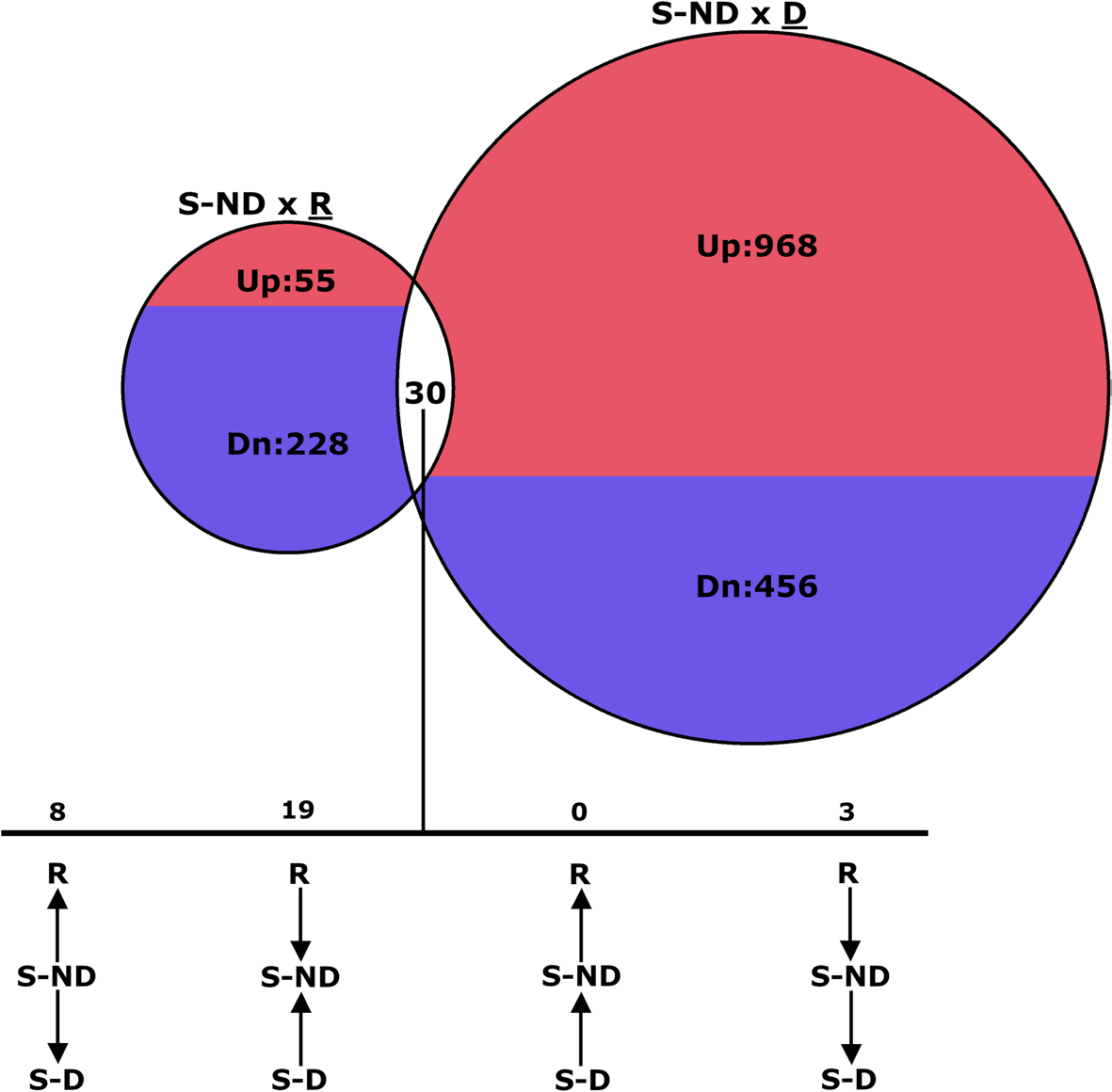
Venn diagram showing number and fold-change type of differentially expressed genes (DEG) for non-diseased susceptible pecan leaves (S-ND) when compared separately to diseased (S-D) and resistant (R) leaves. Treatments compared are above their respective results. Up- (Up) or down-regulation (Dn) describes expression pattern of underlined treatment group, when compared to S-ND. Fold-change patterns for DEGs shared between comparisons is described in detail below Venn diagram with arrows indicating direction of increasing expression

When comparing expression data from S-ND to S-D leaves, 1,454 DEGs were identified (Fig. 3; Additional file 1, tab 3 “DxND_DEGs”).

Of these, 475 genes were down-regulated and 979 genes were up-regulated for S-D compared to S-ND leaves. In contrast, fewer genes were differentially expressed when comparing expression data of R to S-ND leaves (Fig. 3; Additional file 1, tab 4 “RxND_DEGs”). Of the 313 DEGs identified for this comparison, sixty-three were up-regulated and 250 were down-regulated in R compared to S-ND leaves. The two sets of DEGs, S-ND by S-D and S-ND by R, had thirty genes in common.

### Analysis of differentially expressed genes

**Fig. 4:**
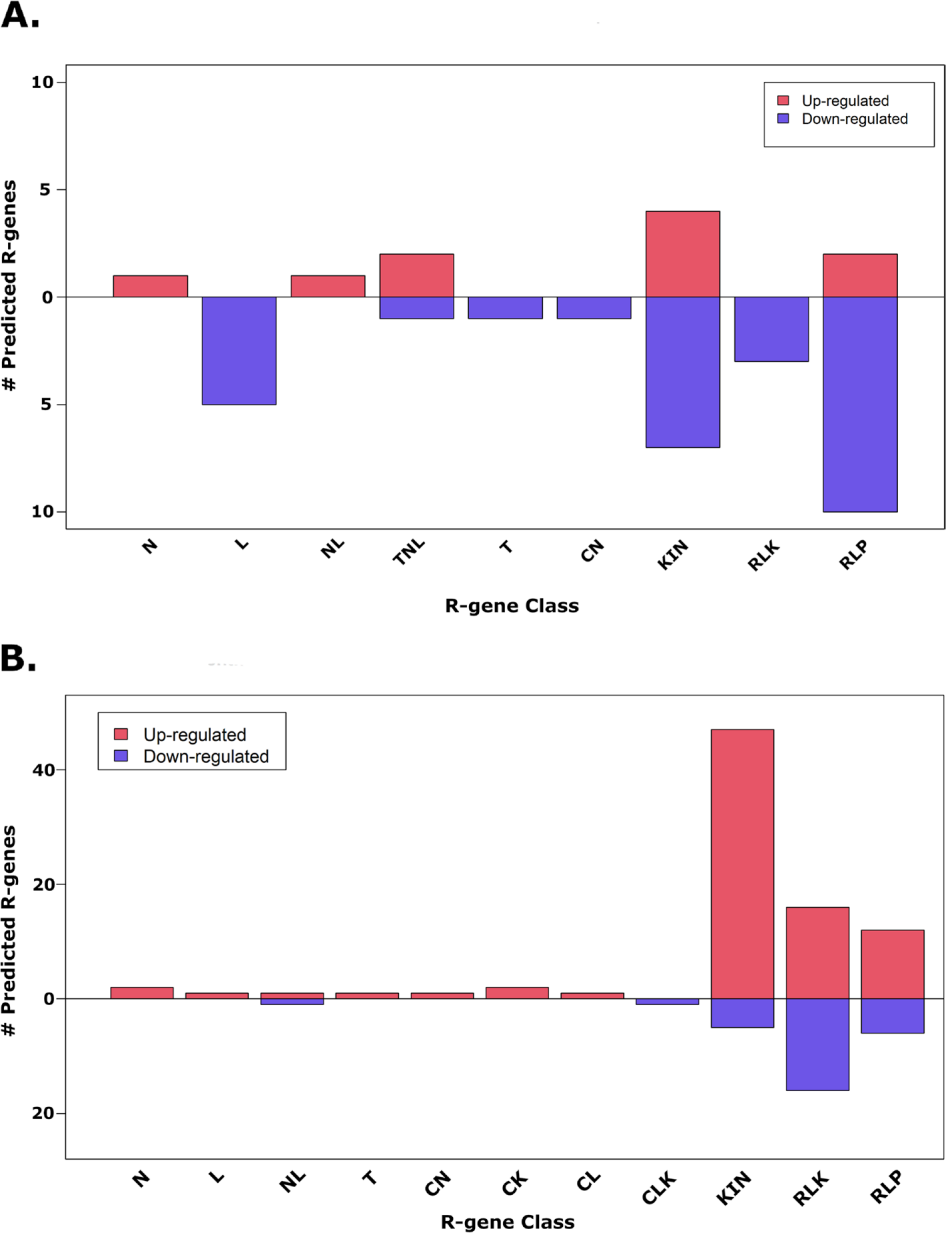
Differentially expressed predicted R-genes for pecan scab related treatments. Regulation type explains fold change for (**A**) resistant leaves (R), and (**B**) diseased leaves (S-D) when compared to susceptible non-diseased leaves (S-ND). Abbreviations: N=nucleotide binding site (NBS), L=leucine rich-repeat (LRR), NL=NBS-LRR, TNL=TIR-NB-LRR, T=toll-interleukin receptor (TIR), CN=coiled-coil (CC)-NBS, CK=CC-kinase, CL=CC-LRR, CLK=CC-LRR-kinase, KIN=kinase, RLK=receptor-like kinase, RLP=receptor like protein

Resistance genes (R-genes) are important components of the immune system plants depend on for resistance to pathogens. The program NLR-annotation was able to identify 345 R-genes from the 31,911 annotated 87MX3-2.11 predicted pecan genes. In contrast, DRAGO was more sensitive, predicting 2,410 R-genes from the same gene set (Additional file 1, tab 5 “DRAGO_rGenes”). This difference could be due to DRAGO using more R-gene motifs for prediction. For example, the number of R-genes containing both NBS and LRR sequences in the DRAGO2 R-gene set was much closer to that of the total NLR-annotation set at 218. Subsequent results are based on DRAGO predictions as it gives more detailed information on R-gene types. Of the 313 DEGs identified for the R by S-ND comparison, thirty-eight were identified as R-genes by DRAGO2, ten of which were up-regulated in R samples (Fig. 4A).

Kinase domain-containing proteins (KIN) were the most up-regulated type of R-gene (four genes), although the same class was more down-regulated (seven genes). The most down-regulated type of R-gene for the R by S-ND comparison were receptor-like proteins (RLP, ten genes). Classes of R-gene containing NB-LRR motifs (NL & TNL) were slightly up-regulated in R samples. For the S-ND by S-D comparison 113 of the DEGs were predicted R-genes, with eighty-three of these being up-regulated for S-D (Fig. 4B). Similar to the R by S-ND comparison, kinase domain containing proteins were the most up-regulated type of R-gene (forty-seven genes) for S-D compared to S-ND; however, relatively few were down-regulated (five genes). The most down-regulated type of R-gene for this comparison were receptor-like kinases (sixteen genes), but this class was up-regulated to the same extent.

**Fig. 5:**
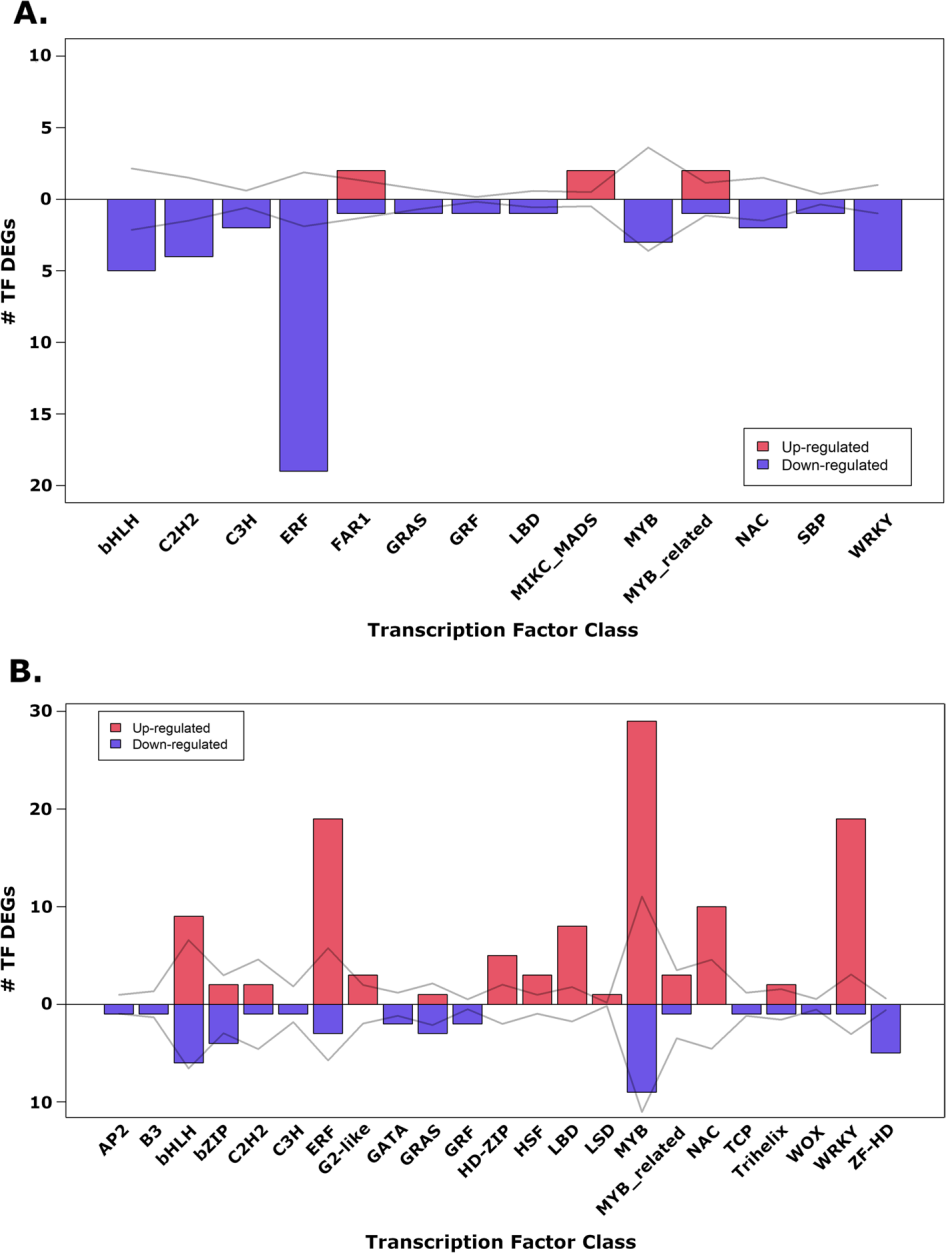
Differentially expressed (DE) transcription factors (TFs) for different pecan scab treatments. Regulation type explains fold change for (**A**) resistant leaves (R), and (**B**) susceptible diseased leaves (S-D) when compared to non-diseased susceptible leaves (S-ND). Line graph shows number of expected DE TFs if chosen at random from all *Carya illinoinensis* TFs as predicted with PlantTFDB v5.0 (1/2 DE TF * ratio *C. illinoinensis* TF class)

Transcription factors (TFs) can regulate the expression of large sets of genes to control complex traits such as pathogen resistance in plants. When evaluating genes annotated for the *C. illinoinensis* 87MX3-2.11 genome, PlantTFDB and DeepTFactor were able to predict 2,233 and 2,547 transcription factors respectively. The following results are based on the PlantTFDB predicted TFs, as its output gives more detailed information on TF type (Additional file 1, tab 6 “PlantTFDB_TFs”). Differential expression of TFs in comparisons evaluated reflected the respective overall differential expression for each of the two comparisons. TFs were generally down-regulated in R compared to S-ND leaves (Fig. 5A), whereas S-D leaves compared to S-ND showed a significant amount of up-regulation in TF expression (Fig. 5B).

The largest discrepancy between the two comparisons was down-regulation in R and up-regulation in S-D of ethylene response factors when compared to S-ND samples. Also, WRKY and MYB type TFs seem to be over-represented in up-regulation for S-D when compared to S-ND.

Enriched gene ontology (GO) analysis and gene set analysis (GSA) are powerful tools for drawing conclusions from large sets of DEGs. For the R by S-ND comparison, fifteen up-regulated genes met requirements for GO analysis and 110 were used for down-regulated DEG analysis. For the S-D by S-ND comparison, 370 up-regulated and 205 down-regulated DEGs were used for GO analysis. GO enrichment was identified for both R by S-ND and S-D by S-ND comparisons. While the S-D by S-ND comparison showed enrichment for each category evaluated (“cellular component”, “molecular function”, and “biological process”) for both down- and up-regulation (Additional file 1, tab 7 “GOrilla_DxND”; Additional file 2, Figures 1 & 2); the R by S-ND comparison only displayed enrichment for down-regulation of “biological process” and up-regulation of “molecular function” (Additional file 1, tab 8 “GOrilla_RxND”; Additional file 2, Figure 3). The most significantly enriched term for R by S-ND was down-regulation of “hormone catabolic process” in R leaves (GO:0042447; *p*-value 6.99e-5). Surprisingly, other enriched down-regulated GO terms in R samples were “defense response to other organism” and “response to chitin”. “Pyrimidine transmembrane transporter activity” was the only enriched term for up-regulation in R samples. The most significantly enriched term for the S-D by S-ND comparison was down-regulation of “cellular component extracellular region” in S-D leaves (GO: GO:0005576; *p*-value 1.38e-8). Surprisingly, the same term had a similar significance for up-regulation in S-D (*p*-value 1.77e-8). Other terms that were found to be enriched for down-regulation in S-D include “endopeptidase activity” and “response to auxin”. GO terms up-regulated in S-D include a myriad of biotic stress responses (excluding “response to fungi”), “ethylene-activated signaling pathway”, and “transcription factor activity”.

**Fig. 6:**
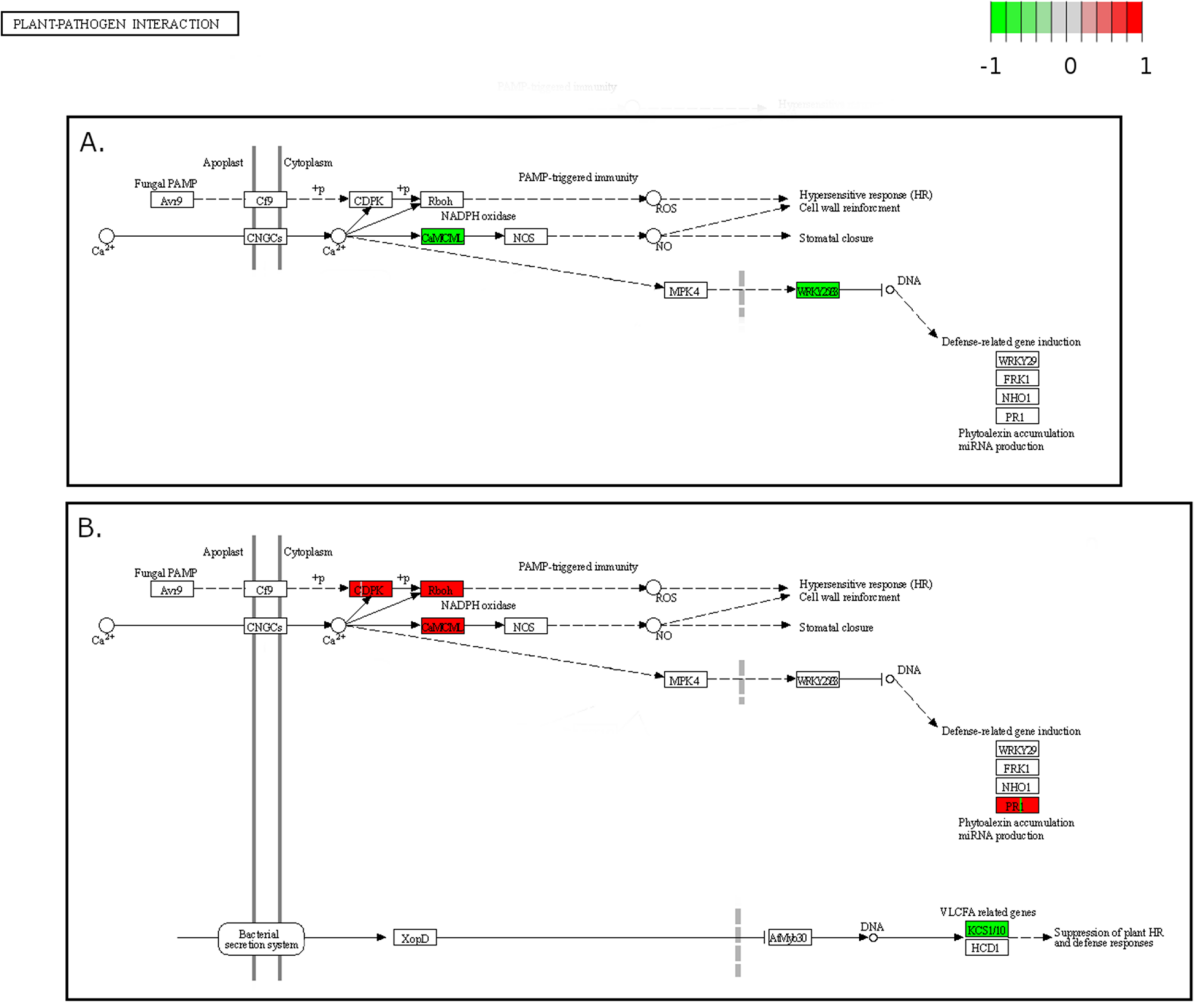
Differentially expressed genes (DEGs) in the “plant-pathogen interaction” KEGG pathway 04626 for (**A**) pecan (*Carya illinoinensis*) trees resistant to *Venturia effusa* (R) as opposed to susceptible non-diseased trees (S-ND) and (**B**) susceptible diseased leaves (S-D) compared to susceptible non-diseased (S-ND) leaves [48]. Significant DEGs have a Benjamini & Hochberg adjusted *p*-value < 0.05 and an absolute log2 fold change > 1.5. Genes in the figure with no expression information are not differentially expressed to a significant extent or *C. illinoinensis* DEGs have no *Arabidopsis thaliana* ortholog information. Pathway element in the figure shows expression pattern for each individual in the case group (R or S-D) compared to S-ND

Gene set analysis with Pathview shows more detailed information on regulation of genes within biochemical pathways compared to GO analysis. For the R by S-ND comparison, the Pathview auto-select function identified three pathways that were enriched for differential expression. These pathways were “RNA degradation” (KEGG pathway 03018), “plant hormone signal transduction” (KEGG pathway 04075), and “plant-pathogen interaction” (KEGG pathway 04626). All pathway terms for this comparison were generally down-regulated in R compared to S-ND. DEGs in the plant-pathogen interactions pathway included WRKY25 and WRKY33 type transcription factors as well as a calcium-binding protein (CML40) (Fig. 6A).

DEGs in the plant-pathogen interaction pathway for S-D were generally up-regulated compared to S-ND samples (Fig. 6B). Pathview detected an element of the Ccr4-NOT complex to be down-regulated in the “RNA degradation” pathway as well as elements of the brassinosteroid and jasmonic acid (JA) pathways in the “plant hormone signaling” pathway. Other elements down-regulated in R samples that were not auto-selected by Pathview include genes responsible for camalexin synthesis, ethylene synthesis, defense responses, and wounding responses. All original Pathview output files can be found in Additional file 2, Figures 4-12.

Unlike the R by S-ND comparison, Pathview showed a mixture of down- and up-regulated enriched gene sets for the S-ND by S-D comparison. The auto-select feature of Pathview reported two pathways, “plant hormone signaling” and the “starch/sucrose metabolism” pathway (KEGG pathway 00500). For the “plant hormone signaling” pathway many genes were down-regulated for auxin signaling in S-D compared to S-ND, up-regulated for gibberellin, ethylene, and jasmonic acid signaling, and perturbed for salicylic acid signaling. For the “starch/sucrose metabolism” pathway, genes were up-regulated in S-D for gene products responsible for the biosynthesis of trehalose, D-glucose-6P, and D-fructose. In contrast, many genes responsible for the production of D-glucose and its intermediates were down-regulated. When examining plant specific pathways using Pathview, genes responsible for plant defenses were consistently up-regulated in S-D leaves. Descriptors for pathways belonging to these genes include “disease resistance”, “hypersensitive response (HR)”, “defense-related gene induction”, and “late defense responses for pathogens”. In addition to this, elements responsible for suppression of plant HR and suppression of defense responses were down-regulated.

## Discussion

*Venturia effusa* is a formidable fungal pathogen of pecan. Genetic resistance to this phytopathogen occurs naturally in some pecan trees and is complicated by the presence of different pathovars. Breeding for resistance can be slow due to the prolonged time required for pecan to begin flowering and setting fruit. The research presented here aims to decipher which genetic elements are responsible for resistance/susceptibility to *V. effusa* on the basis of gene expression. To meet this goal, the current study leverages the comprehensive genetic diversity present in the pecan provenance collection at Byron, GA, along with years of pecan scab phenotyping data against diverse natural populations of *V. effusa*. The trees evaluated in this study cover a range of the genetic diversity of *C. illinoinensis* as they represent several of the provenances in the collection (Additional file 2, Figure 13), which itself accounts for 94% of the genetic diversity of the species [3].

**Fig. 7:**
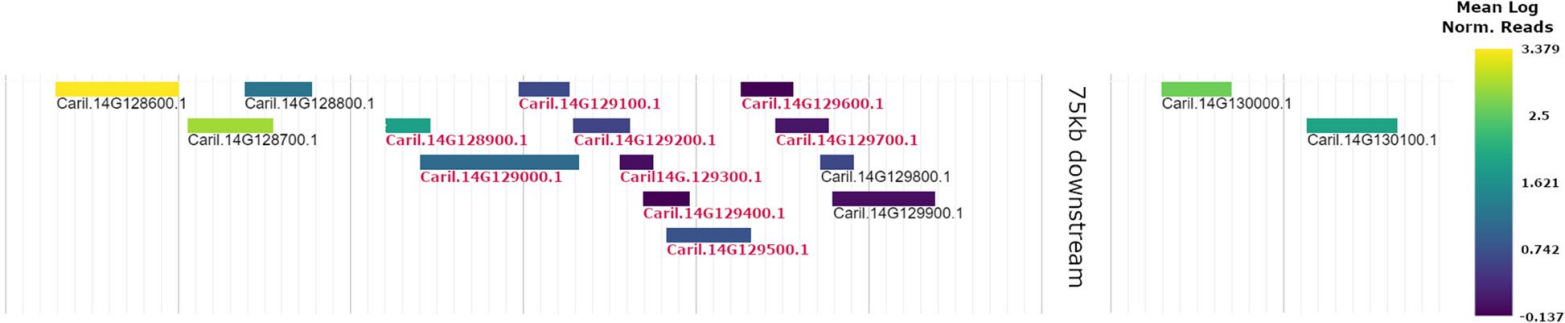
Gene expression at predicted susceptibility quantitative trait loci (QTL) and flanking regions in scab resistant leaves of pecan (*Carya illinoinensis* 87MX3-2.11). Red gene names indicate genes that are significantly down-regulated. Eight of the nine differentially expressed genes (DEGs) are predicted leucine rich repeat R-genes. Expression of most proximal 5’ and 3’ genes also shown

Each of the conditions examined, S-ND, S-D, and R leaves, showed unique expression patterns. These expression patterns are essential for gaining insight into different aspects of the pecan scab pathosystem. By comparing R and S-ND samples one might elucidate factors responsible for preventing or allowing initial infection by *V. effusa*. Whereas comparing S-ND to S-D, potential targets of fungal effectors may be identified, among other genes active in the infection process. The comparison R to S-D was not evaluated, as it confounds diseased state and basal expression type factors. The larger proportion of down-regulated DEGs in R samples could indicate susceptibility factors as a causal agent of resistance/susceptibility to pecan scab. When examining the distribution of differentially expressed R-genes within the 87MX3-2.11 genome, one locus contained eight down-regulated leucine rich-repeat type predicted R-genes (Fig. 7, Additional file 1; tab 9 “SuscQTL”).

If population level resistance to pecan scab is dependent on R-gene mediated resistance however, a single up-regulated R-gene could be responsible for resistance despite the general down-regulation of R-genes in resistant samples. Regardless, expression of each of the up-regulated DEG predicted R-genes in resistant samples is present in S-ND samples. Their presence in susceptible samples should negate them as possible sources of resistance, at least at the level of gene expression. This is because R-gene mediated resistance in plants typically shows a well-defined dominance-recessive phenotype.

A number of other essential components of plant defense pathways are also down-regulated in resistant trees. Mitogen-activated protein kinases (MAPK) are intermediate elements of the defense pathway, carrying signals generated by R-genes to the nucleus resulting in the expression of a myriad of defense genes (Reviewed in: [53]). Genes set analysis was able to identify MPK9 as being down-regulated in the R group. As a further point of confusion, despite WRKY33 being shown to be essential for resistance to fungal pathogens in *A. thaliana* [54], WRKY33 is down-regulated in scab resistant pecan. Regardless of the down-regulation for these upstream genes of the defense pathway, there was no significant difference in expression of known defense genes such as PR1 in resistant trees compared to susceptible trees.

Potentially less obvious components of the plant immune system are also down-regulated in resistant pecan trees. A component of the CCR4-CAF1-Not1 cytoplasmic deadenylase complex, CNOT7/8, is down-regulated. In Arabidopsis, over-expression of another component of this complex led to increased susceptibility to the necrotrophic fungal pathogen *Botrytis cinerea* [55]. Of the few genes up-regulated in resistant pecan samples, gene ontology enrichment identified up-regulation of uracil transmembrane transporter activity. Rapidly growing plant tissues, like the expanding leaves required for *V. effusa* infection, are a sink for a variety of biomolecules. Since uracil is an intermediate for important enzyme cofactors, signaling molecules, and secondary metabolites, the up-regulation of uracil transporter activity in resistant plants could allow them to respond more quickly to *V. effusa* in a tailored fashion. Data analyzed here could be compared to a closer related plant species with a similar pathogen such as apple (*Malus domestica*) and apple scab (*Venturia inaequalis*); although, there are obvious limitations with non-model systems. For example, *A. thaliana* has forty-two terms of its KEGG plant-pathogen interaction pathway defined while apple only has thirty-seven terms defined.

In stark contrast to the R by S-ND comparison, DEGs in S-D leaf samples were largely up-regulated when compared to S-ND leaf samples. Enriched gene ontology analysis identified similar terms compared to the cultivar-pathovar level analysis done by Lovell et al. [33]. These terms include down-regulation of “extracellular region” (GO:0005576), down-regulation of “response to auxin” (GO:0009733), and up-regulation of “DNA-binding transcription factor activity” (GO:0003700). Expression patterns for components of plant defense pathways clearly show recognition of the pathogen by susceptible plants. For example, two fungal/ethylene inducible genes PR-1 and ChiB were consistently up-regulated [56, 57]. Also, jasmonic acid/ethylene signaling pathways are used by plants for resistance to fungal necrotrophs [58, 59]. Genes in both of these pathways were identified to be significantly up-regulated in susceptible diseased samples. Resolution of the current study is not detailed enough to determine if these defenses are responsible for disease susceptibility or are successful mechanisms for preventing further spread of infection to healthy plant tissues. Deliberately triggering host defenses is a well-documented infection strategy for necrotrophic fungal phytopathogens [60, 61]. If on the other hand these defenses are responsible for stopping the spread of the pathogen to healthy tissues, they represent prime candidates for overexpression lines for decreasing disease severity to *V. effusa*. RNA-seq experiments using controlled inoculations, time-course series, and laser-capture microdissection are better suited to answer these questions.

Dissimilar transcriptome profiles are the underlying cause for cellular, tissue, and structural differences found within the same individual. The correlation between scab susceptibility in fruit and leaf tissue is high [4]. Given this, genes and pathways responsible for resistance to diverse populations of *V. effusa* could be further refined by comparing fruit whole transcriptome profiles of the trees in this study to their counterpart leaf data. Evaluating the proteomics and metabolomics in these trees will also help give a better understanding of the intricacies of the pecan/scab pathosystem. Because S-ND and S-D samples were collected as pairs from each susceptible tree, these samples could be analyzed as paired-data. Analysis performed using this method has been shown to have increased power at detecting differentially expressed genes that have lower average expression and shorter transcript length [62]. Since *V. effusa* was present on the same tree that S-ND samples were collected from, long-range transport and signaling of phytohormones could skew S-ND samples toward an expression profile more closely resembling S-D. This has the potential to produce artifacts in both the S-ND by S-D and R by S-ND comparisons made in this study. To mitigate for this, a combination of fungicides, metabolomics, and time-course sampling could be performed in future studies. Despite the uncertainties present in the S-ND treatment group, controlled inoculations of susceptible pecan done by Lovell et al. (2021) gave similar gene ontology enrichment results as those obtained for the S-ND by S-D comparison done for the present study. This would suggest that the S-ND samples do indeed reflect basal expression of susceptible plants. Analysis of basal expression for resistant and susceptible plants is often overlooked despite the known importance of basal defenses and susceptibility factors (Reviewed in: [63, 64]). Including this type of analysis when examining other pathosystems will help draw parallels and explain the importance of these factors in pathogen resistance/susceptibility. Furthermore, a large portion of the predicted *C. illinoinensis* genes have missing *A. thaliana* and GO descriptions (34.8% and 38.0% respectively for the Phytozome 573 v1.1 annotation). As our understanding of gene functions continues to grow, gene ontology and gene set analysis will become more powerful at shedding light on this important pecan pathosystem.

## Conclusions

The present study shows differentially expressed genes of scab resistant pecan trees to be generally down-regulated while susceptible pecan trees have many up-regulated defense related pathways upon infection. This makes a compelling case for susceptibility as the basis for compatibility between the fungus *V. effusa* and pecan. Differentially expressed genes identified by this study will aid further investigation in the *V. effusa/*pecan interaction and provide a means for ultimately developing durable resistance to this formidable pathogen.

### Supplementary Information

**Additional file 1. **Differentially expressed genes plus other supporting information. Differentially expressed genes including annotation information, predicted* Carya illinoinensis *transcription factors, predicted* Carya illinoinensis *pathogen resistance genes, GOrilla gene ontology enrichment information, primer information, and susceptibility quantitative trait loci information.

**Additional file 2:** **Figure 1.** Gene ontology enrichment results from GOrilla reporting significantly down-regulated ontology types in susceptible diseased samples (S-D), compared to susceptible non-diseased samples (S-ND). Significance of GO term is indicated with the color corresponding to the *P*-value color scale at the top of the figure. **Figure 2.** Gene ontology enrichment results from GOrilla reporting significantly up-regulated ontology types in susceptible diseased samples (S-D), compared to susceptible non-diseased samples (S-ND). Significance of GO term is indicated with the color corresponding to the *P*-value color scale at the top of the figure. **Figure 3.** Gene ontology enrichment results from GOrilla reporting down-regulated & up-regulated ontology types in resistant samples (R), compared to susceptible non-diseased samples (S-ND). Significance of GO term is indicated with the color corresponding to the*P*-value color scale at the top of the figure. Ontology pathway-regulation types not shown were not reported by GOrilla. **Figure 4.** Kegg pathway (00500) analysis via Pathview for differentially expressed genes (DEG; absolute log_2_ fold change > 1.5, Benjamini & Hochberg adjusted *p*-value less than 0.05) in susceptible-diseased samples (S-D), compared to susceptible non-diseased samples (S-ND). Color represents expression pattern. Genes without expression information were either not differentially expressed or *Carya illinoinensis* DEGs had no corresponding *Arabidopsis thaliana* ortholog information. Expression pattern for each case sample (S-D) is represented in each gene box. Reproduction of KEGG pathway granted by Kanehisa Laboratories under open access license. **Figure 5.** Kegg pathway (00999) analysis via Pathview for differentially expressed genes (DEG; absolute log_2_ fold change > 1.5, Benjamini & Hochberg adjusted *p*-value less than 0.05) in susceptible-diseased samples (S-D), compared to susceptible non-diseased samples (S-ND). Color represents expression pattern. Genes without expression information were either not differentially expressed or *Carya illinoinensis* DEGs had no corresponding *Arabidopsis thaliana* ortholog information. Expression pattern for each case sample (S-D) is represented in each gene box. Reproduction of KEGG pathway granted by Kanehisa Laboratories under open access license. **Figure 6.** Kegg pathway (04016) analysis via Pathview for differentially expressed genes (DEG; absolute log_2_ fold change > 1.5, Benjamini & Hochberg adjusted *p*-value less than 0.05) in susceptible-diseased samples (S-D), compared to susceptible non-diseased samples (S-ND). Color represents expression pattern. Genes without expression information were either not differentially expressed or *Carya illinoinensis* DEGs had no corresponding *Arabidopsis thaliana* ortholog information. Expression pattern for each case sample (S-D) is represented in each gene box. Reproduction of KEGG pathway granted by Kanehisa Laboratories under open access license. **Figure 7.** Kegg pathway (04075) analysis via Pathview for differentially expressed genes (DEG; absolute log_2_ fold change > 1.5, Benjamini & Hochberg adjusted *p*-value less than 0.05) in susceptible-diseased samples (S-D), compared to susceptible non-diseased samples (S-ND). Color represents expression pattern. Genes without expression information were either not differentially expressed or *Carya illinoinensis* DEGs had no corresponding *Arabidopsis thaliana* ortholog information. Expression pattern for each case sample (S-D) is represented in each gene box. Reproduction of KEGG pathway granted by Kanehisa Laboratories under open access license. **Figure 8.** Original Kegg pathway (04626) analysis via Pathview for differentially expressed genes (DEG; absolute log_2_ fold change > 1.5, Benjamini & Hochberg adjusted *p*-value less than 0.05) in susceptible-diseased samples (S-D), compared to susceptible non-diseased samples (S-ND). Color represents expression pattern. Genes without expression information were either not differentially expressed or *Carya illinoinensis* DEGs had no corresponding *Arabidopsis thaliana* ortholog information. Expression pattern for each case sample (R) is represented in each gene box. Reproduction of KEGG pathway granted by Kanehisa Laboratories under open access license. **Figure 9.** Kegg pathway (03018) analysis via Pathview for differentially expressed genes (DEG; absolute log_2_ fold change > 1.5, Benjamini & Hochberg adjusted *p*-value less than 0.05) in resistant samples (R), compared to susceptible non-diseased samples (S-ND). Color represents expression pattern. Genes without expression information were either not differentially expressed or *Carya illinoinensis* DEGs had no corresponding *Arabidopsis thaliana* ortholog information. Expression pattern for each case sample (R) is represented in each gene box. Reproduction of KEGG pathway granted by Kanehisa Laboratories under open access license. **Figure 10.** Kegg pathway (04016) analysis via Pathview for differentially expressed genes (DEG; absolute log_2_ fold change > 1.5, Benjamini & Hochberg adjusted *p*-value less than 0.05) in resistant samples (R), compared to susceptible non-diseased samples (S-ND). Color represents expression pattern. Genes without expression information were either not differentially expressed or *Carya illinoinensis* DEGs had no corresponding *Arabidopsis thaliana* ortholog information. Expression pattern for each case sample (R) is represented in each gene box. Reproduction of KEGG pathway granted by Kanehisa Laboratories under open access license. **Figure 11.** Kegg pathway (04075) analysis via Pathview for differentially expressed genes (DEG; absolute log_2_ fold change > 1.5, Benjamini & Hochberg adjusted *p*-value less than 0.05) in resistant samples (R), compared to susceptible non-diseased samples (S-ND). Color represents expression pattern. Genes without expression information were either not differentially expressed or *Carya illinoinensis* DEGs had no corresponding *Arabidopsis thaliana* ortholog information. Expression pattern for each case sample (R) is represented in each gene box. Reproduction of KEGG pathway granted by Kanehisa Laboratories under open access license. **Figure 12.** Original Kegg pathway (04626) analysis via Pathview for differentially expressed genes (DEG; absolute log_2_ fold change > 1.5, Benjamini & Hochberg adjusted *p*-value less than 0.05) in resistant samples (R), compared to susceptible non-diseased samples (S-ND). Color represents expression pattern. Genes without expression information were either not differentially expressed or *Carya illinoinensis* DEGs had no corresponding *Arabidopsis thaliana* ortholog information. Expression pattern for each case sample (R) is represented in each gene box. Reproduction of KEGG pathway granted by Kanehisa Laboratories under open access license. **Figure 13.** Principal component analysis showing the genetic diversity of 835 trees of the pecan provenance collection in Byron, GA using genotype by sequencing (data not shown). Scab susceptible and resistant individuals used in the present study are shown as well as Mexico and Texas genotype clustering. Cultivars developed from northern provenance trees (not Mexico or Texas germplasms) are generally considered to be more resistant to pecan scab.

## Data Availability

The dataset supporting the conclusion of this article are available in the National Center for Biotechnology’s Sequence Read Archive (NCBI-SRA) repository, as Bioproject PRJNA992068.
