# Supplementary material for: Transcriptome profile of pecan scab resistant and susceptible trees from a pecan provenance collection
Source: BMC Genomics. 2024 Feb 15;25:180. doi: 10.1186/s12864-024-10010-0 (PMC10868059; doi:10.1186/s12864-024-10010-0)
Supplement: Supplementary file 2 — Additional file 2: Figure 1. Gene ontology enrichment results from GOrilla reporting significantly down-regulated ontology types in susceptible diseased samples (S-D), compared to susceptible non-diseased samples (S-ND). Significance of GO term is indicated with the color corresponding to the P-value color scale at the top of the figure. Figure 2. Gene ontology enrichment results from GOrilla reporting significantly up-regulated ontology types in susceptible diseased samples (S-D), compared to susceptible non-diseased samples (S-ND). Significance of GO term is indicated with the color corresponding to the P-value color scale at the top of the figure. Figure 3. Gene ontology enrichment results from GOrilla reporting down-regulated & up-regulated ontology types in resistant samples (R), compared to susceptible non-diseased samples (S-ND). Significance of GO term is indicated with the color corresponding to theP-value color scale at the top of the figure. Ontology pathway-regulation types not shown were not reported by GOrilla. Figure 4. Kegg pathway (00500) analysis via Pathview for differentially expressed genes (DEG; absolute log2 fold change > 1.5, Benjamini & Hochberg adjusted p-value less than 0.05) in susceptible-diseased samples (S-D), compared to susceptible non-diseased samples (S-ND). Color represents expression pattern. Genes without expression information were either not differentially expressed or Carya illinoinensis DEGs had no corresponding Arabidopsis thaliana ortholog information. Expression pattern for each case sample (S-D) is represented in each gene box. Reproduction of KEGG pathway granted by Kanehisa Laboratories under open access license. Figure 5. Kegg pathway (00999) analysis via Pathview for differentially expressed genes (DEG; absolute log2 fold change > 1.5, Benjamini & Hochberg adjusted p-value less than 0.05) in susceptible-diseased samples (S-D), compared to susceptible non-diseased samples (S-ND). Color represents expression pattern. G [file 12864_2024_10010_MOESM2_ESM.docx]

Additional file 2. Additional_file_2. Supplementary imagery: GOrilla figures, Pathview KEGG pathways original ouput, and principal component analysis of Byron, GA provenance genotype information.


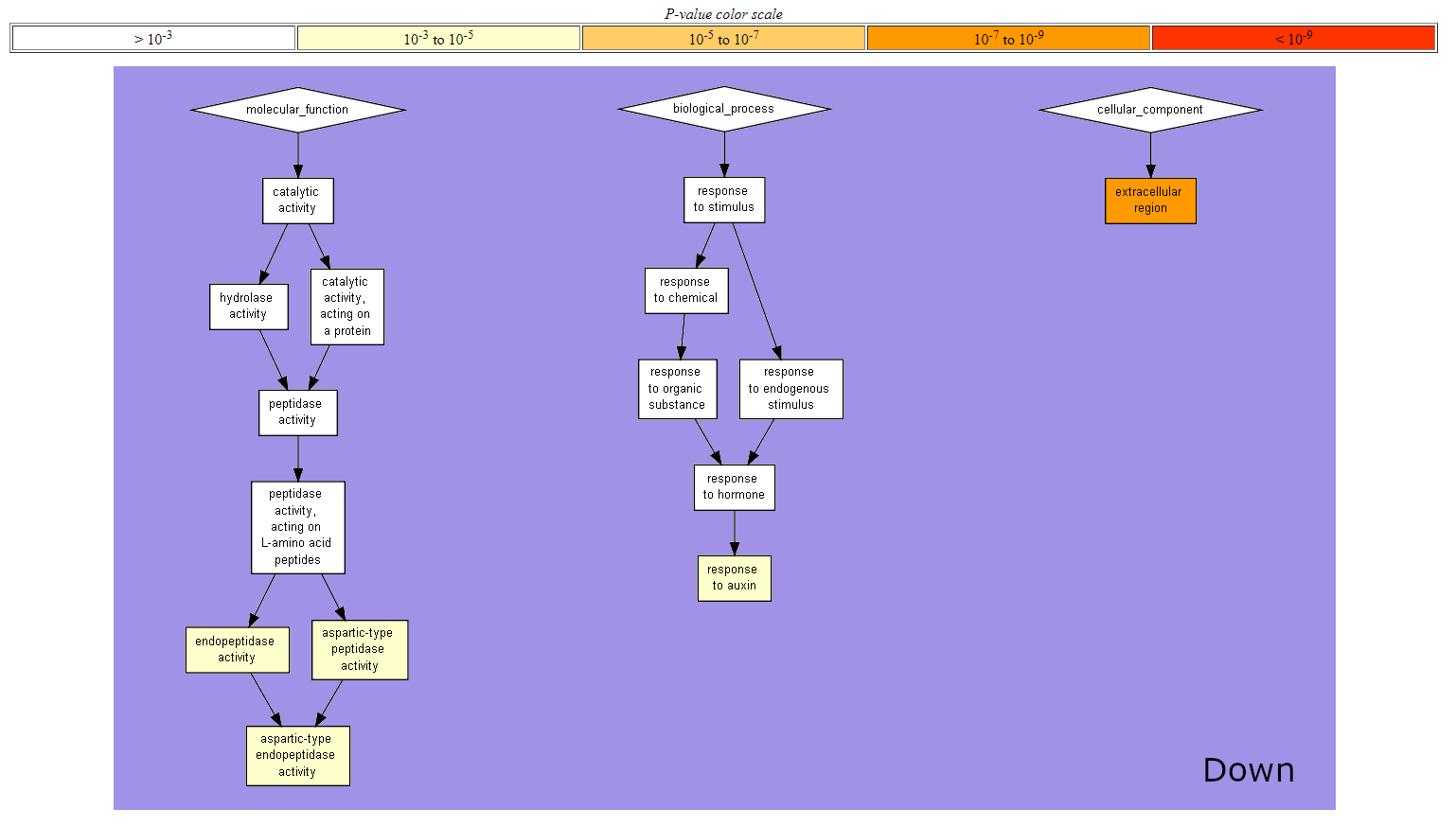


Additional File, Figure 1. Gene ontology enrichment results from GOrilla reporting significantly down-regulated ontology types in susceptible diseased samples (S-D), compared to susceptible non-diseased samples (S-ND). Significance of GO term is indicated with the color corresponding to the P-value color scale at the top of the figure.


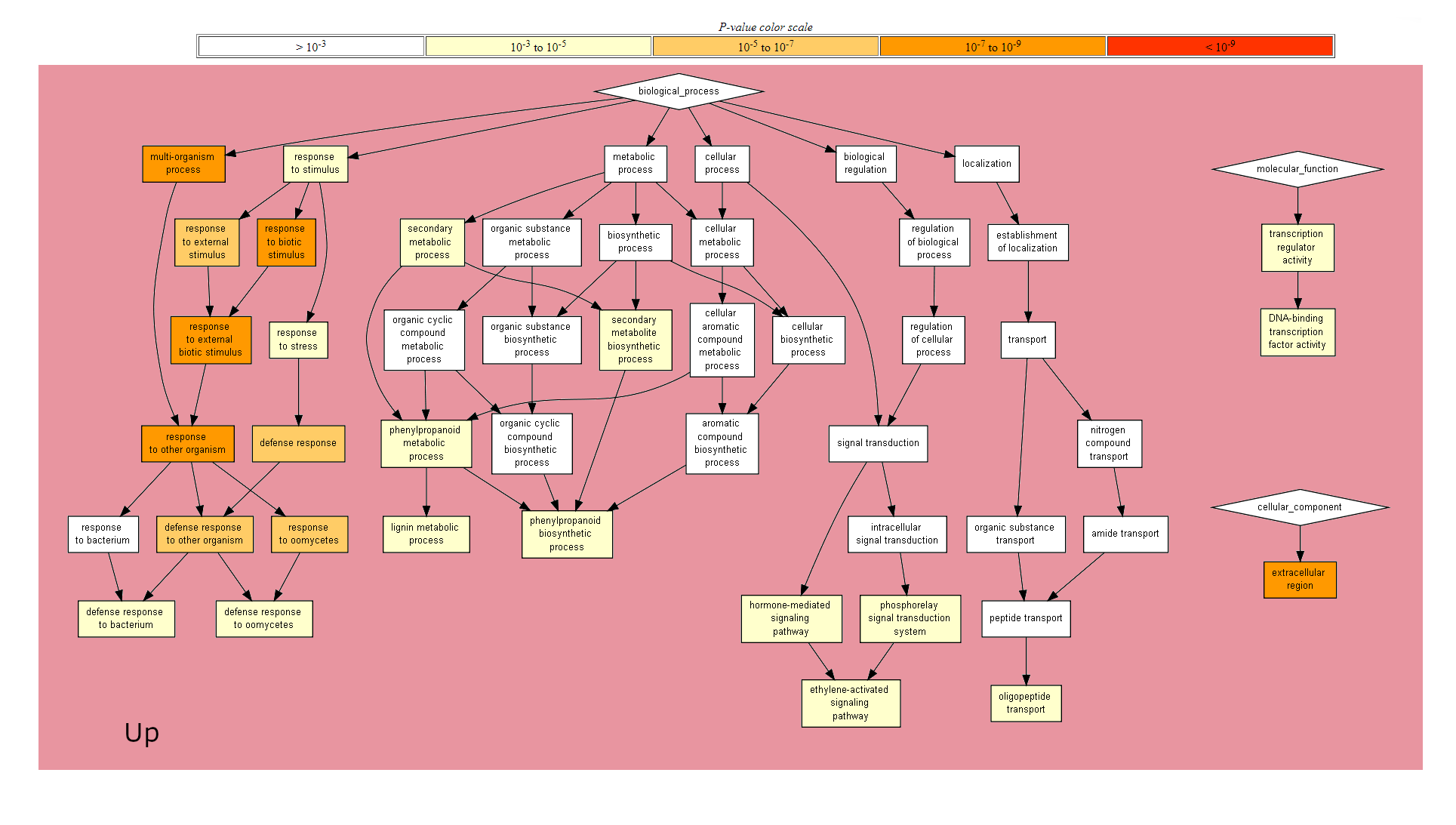


Additional File, Figure 2. Gene ontology enrichment results from GOrilla reporting significantly up-regulated ontology types in susceptible diseased samples (S-D), compared to susceptible non-diseased samples (S-ND). Significance of GO term is indicated with the color corresponding to the P-value color scale at the top of the figure.


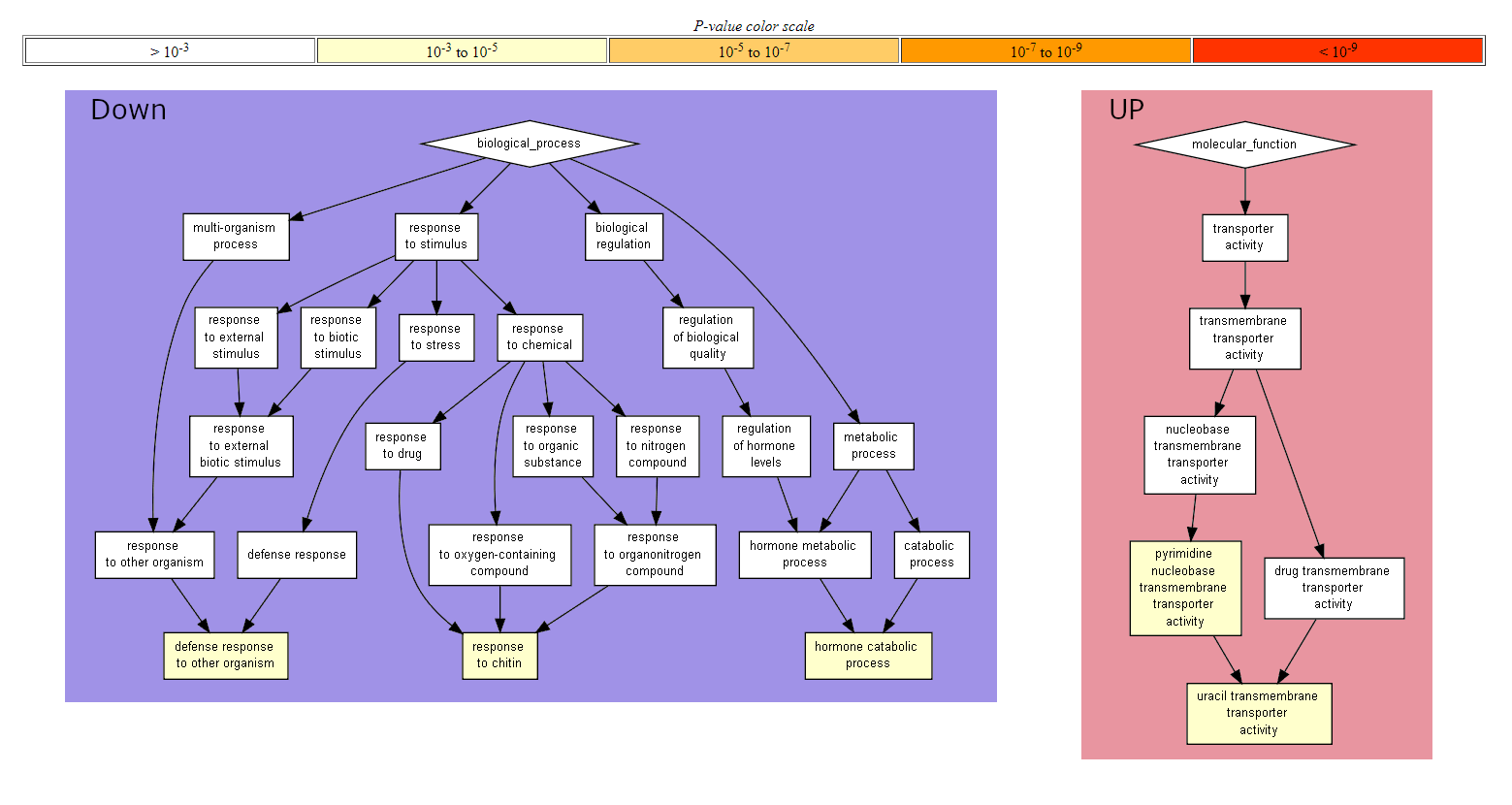


Additional File, Figure 3. Gene ontology enrichment results from GOrilla reporting down-regulated & up-regulated ontology types in resistant samples (R), compared to susceptible non-diseased samples (S-ND). Significance of GO term is indicated with the color corresponding to the P-value color scale at the top of the figure. Ontology pathway-regulation types not shown were not reported by GOrilla.


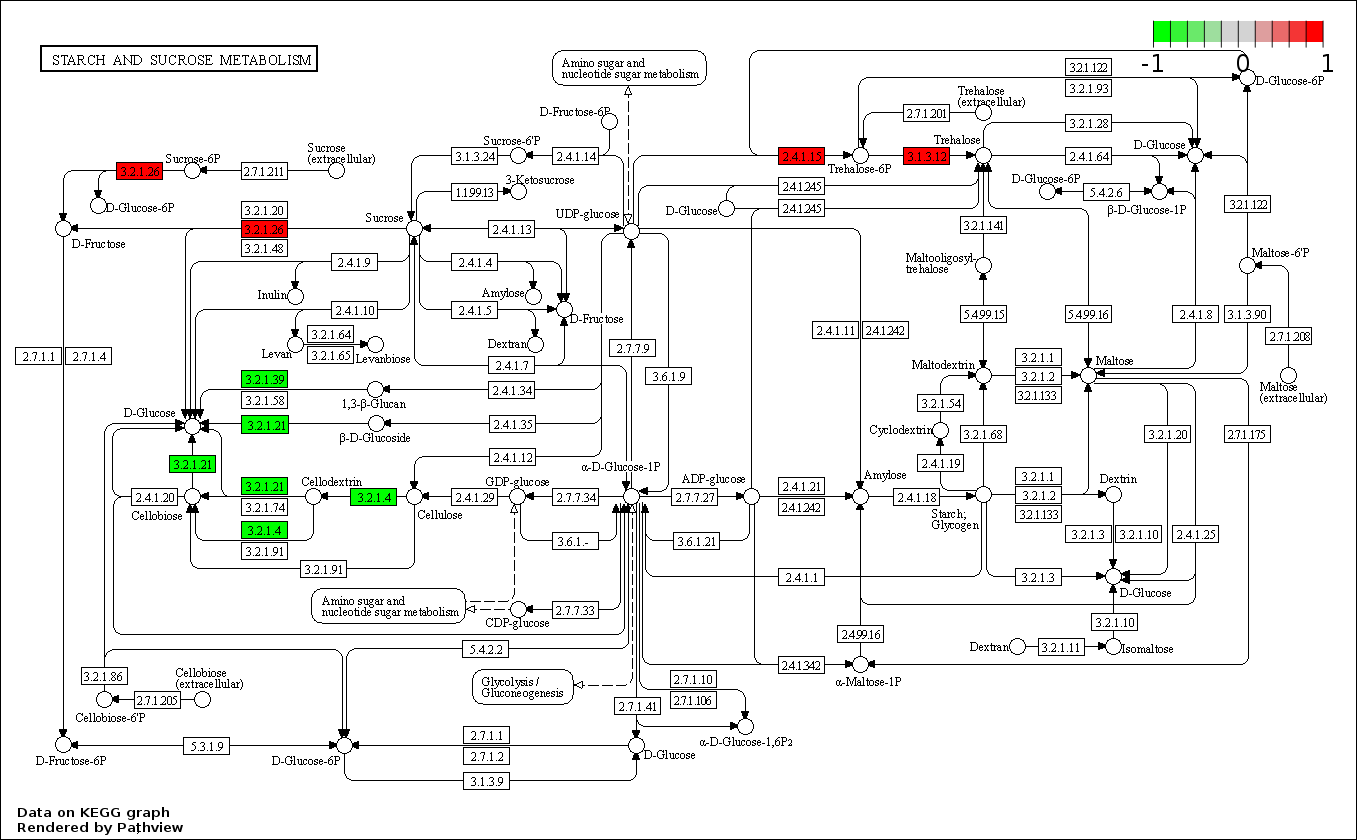


Additional File, Figure 4. Kegg pathway (00500) analysis via Pathview for differentially expressed genes (DEG; absolute log_2_ fold change > 1.5, Benjamini & Hochberg adjusted p-value less than 0.05) in susceptible-diseased samples (S-D), compared to susceptible non-diseased samples (S-ND). Color represents expression pattern. Genes without expression information were either not differentially expressed or *Carya illinoinensis* DEGs had no corresponding *Arabidopsis thaliana* ortholog information. Expression pattern for each case sample (S-D) is represented in each gene box. Reproduction of KEGG pathway granted by Kanehisa Laboratories under open access license.


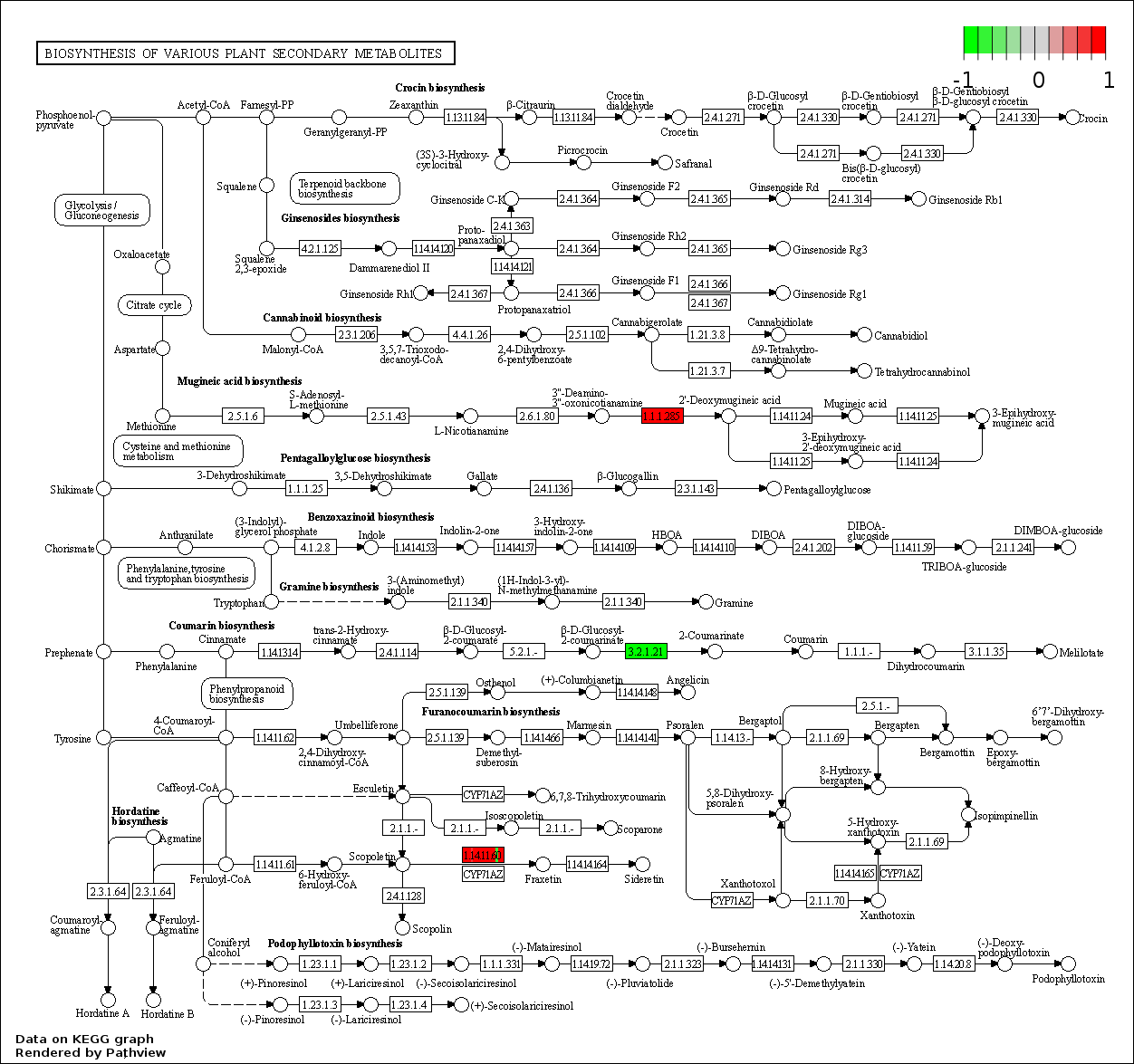


Additional File, Figure 5. Kegg pathway (00999) analysis via Pathview for differentially expressed genes (DEG; absolute log_2_ fold change > 1.5, Benjamini & Hochberg adjusted p-value less than 0.05) in susceptible-diseased samples (S-D), compared to susceptible non-diseased samples (S-ND). Color represents expression pattern. Genes without expression information were either not differentially expressed or *Carya illinoinensis* DEGs had no corresponding *Arabidopsis thaliana* ortholog information. Expression pattern for each case sample (S-D) is represented in each gene box. Reproduction of KEGG pathway granted by Kanehisa Laboratories under open access license.


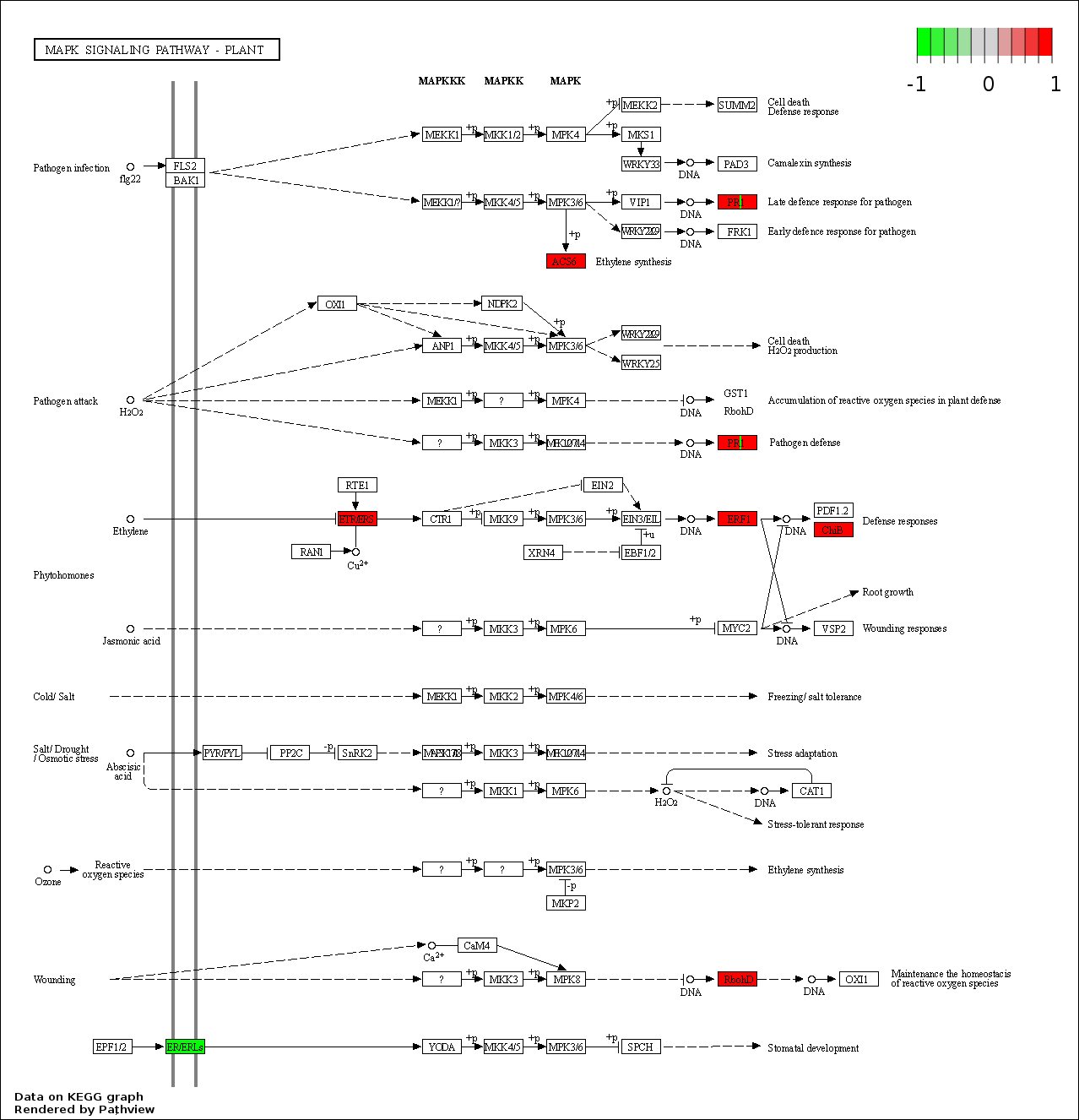


Additional File, Figure 6. Kegg pathway (04016) analysis via Pathview for differentially expressed genes (DEG; absolute log_2_ fold change > 1.5, Benjamini & Hochberg adjusted p-value less than 0.05) in susceptible-diseased samples (S-D), compared to susceptible non-diseased samples (S-ND). Color represents expression pattern. Genes without expression information were either not differentially expressed or *Carya illinoinensis* DEGs had no corresponding *Arabidopsis thaliana* ortholog information. Expression pattern for each case sample (S-D) is represented in each gene box. Reproduction of KEGG pathway granted by Kanehisa Laboratories under open access license.


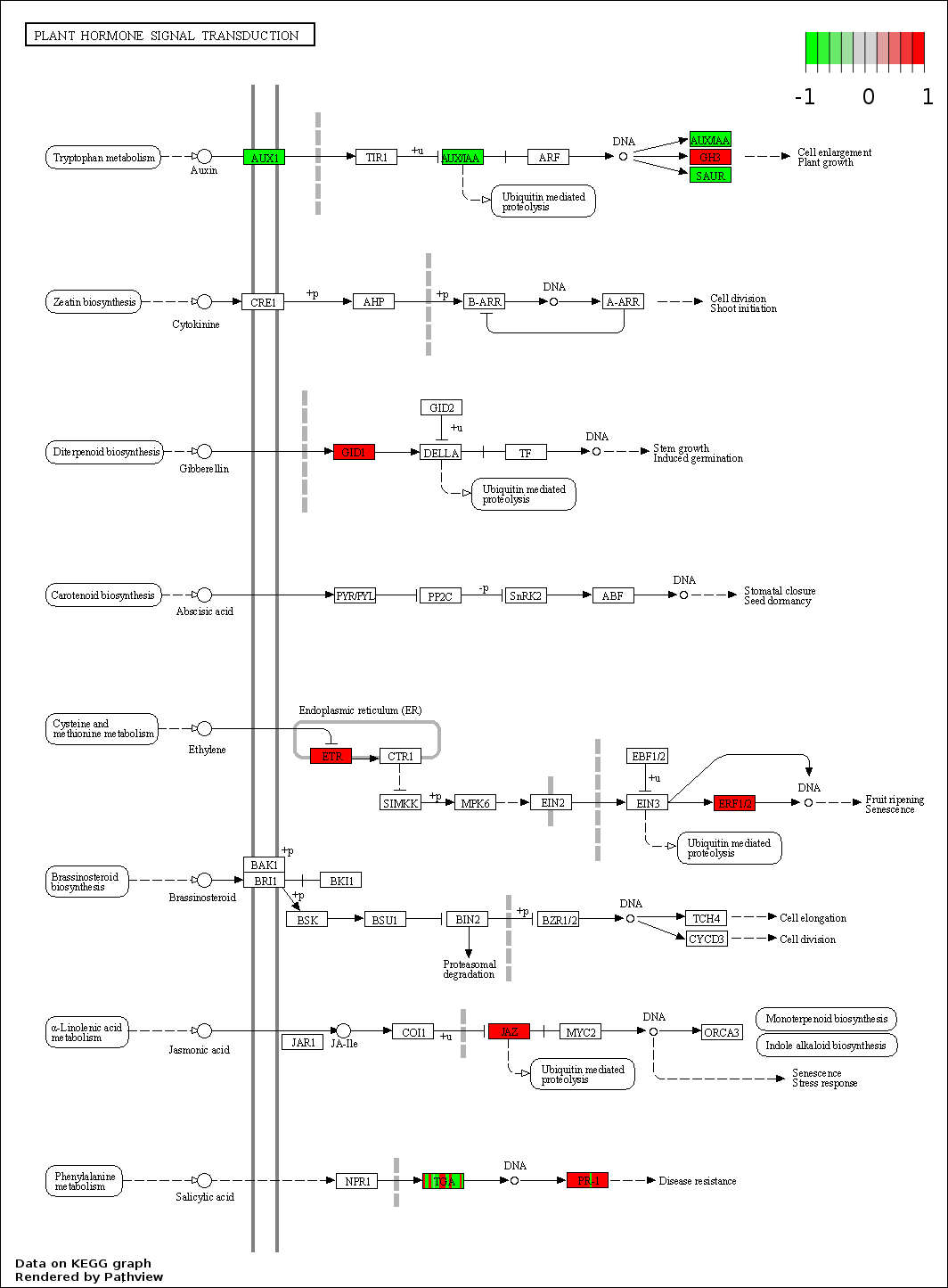


Additional File, Figure 7. Kegg pathway (04075) analysis via Pathview for differentially expressed genes (DEG; absolute log_2_ fold change > 1.5, Benjamini & Hochberg adjusted p-value less than 0.05) in susceptible-diseased samples (S-D), compared to susceptible non-diseased samples (S-ND). Color represents expression pattern. Genes without expression information were either not differentially expressed or *Carya illinoinensis* DEGs had no corresponding *Arabidopsis thaliana* ortholog information. Expression pattern for each case sample (S-D) is represented in each gene box. Reproduction of KEGG pathway granted by Kanehisa Laboratories under open access license.


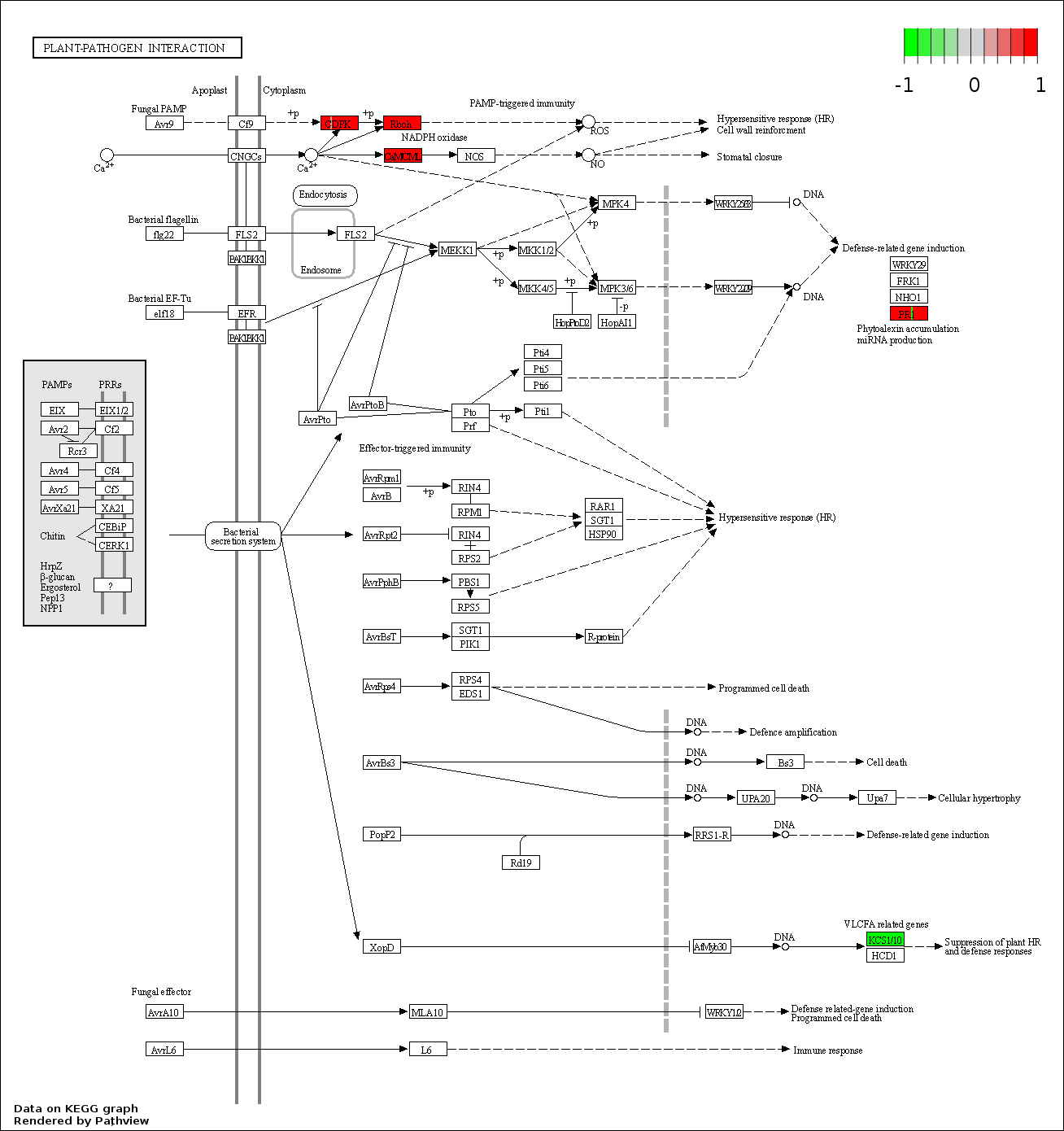


Additional File, Figure 8. Original Kegg pathway (04626) analysis via Pathview for differentially expressed genes (DEG; absolute log_2_ fold change > 1.5, Benjamini & Hochberg adjusted p-value less than 0.05) in susceptible-diseased samples (S-D), compared to susceptible non-diseased samples (S-ND). Color represents expression pattern. Genes without expression information were either not differentially expressed or *Carya illinoinensis* DEGs had no corresponding *Arabidopsis thaliana* ortholog information. Expression pattern for each case sample (R) is represented in each gene box. Reproduction of KEGG pathway granted by Kanehisa Laboratories under open access license.


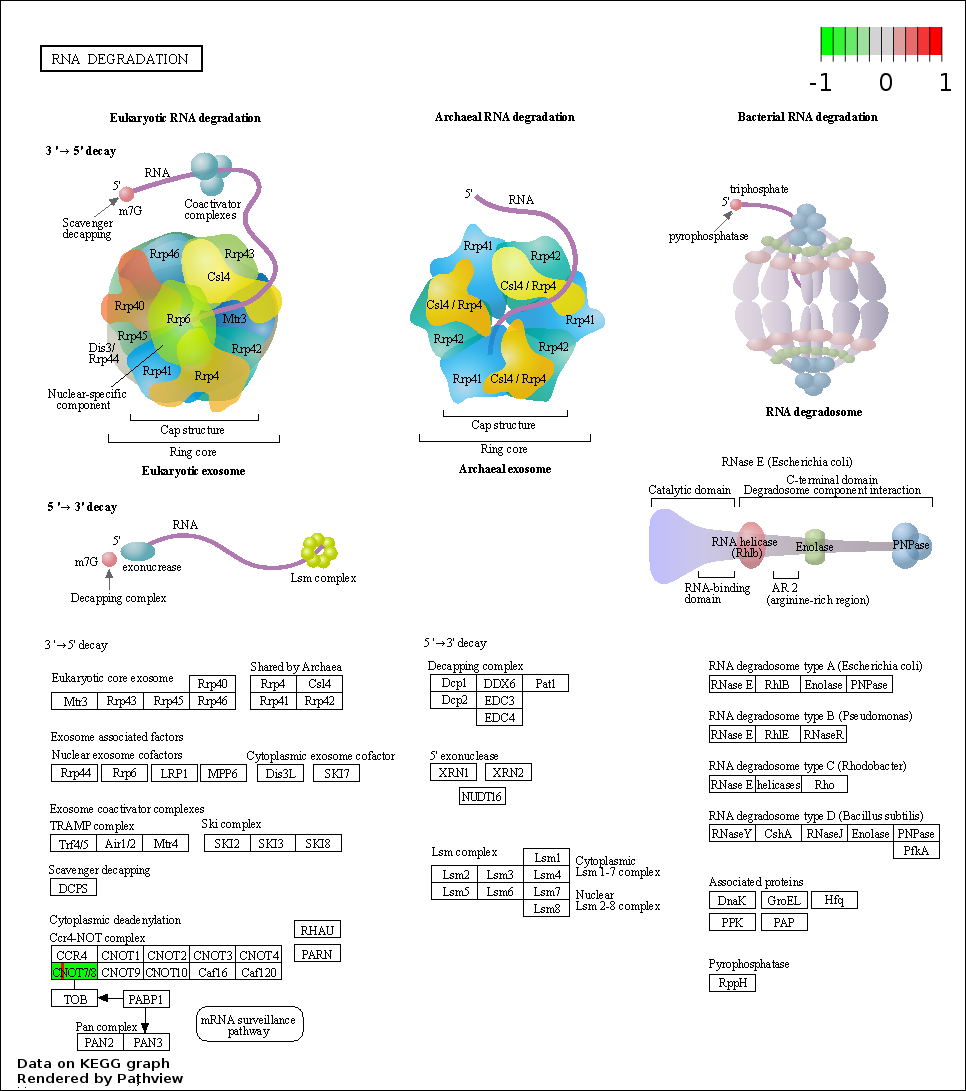


Additional File, Figure 9. Kegg pathway (03018) analysis via Pathview for differentially expressed genes (DEG; absolute log_2_ fold change > 1.5, Benjamini & Hochberg adjusted p-value less than 0.05) in resistant samples (R), compared to susceptible non-diseased samples (S-ND). Color represents expression pattern. Genes without expression information were either not differentially expressed or *Carya illinoinensis* DEGs had no corresponding *Arabidopsis thaliana* ortholog information. Expression pattern for each case sample (R) is represented in each gene box. Reproduction of KEGG pathway granted by Kanehisa Laboratories under open access license.


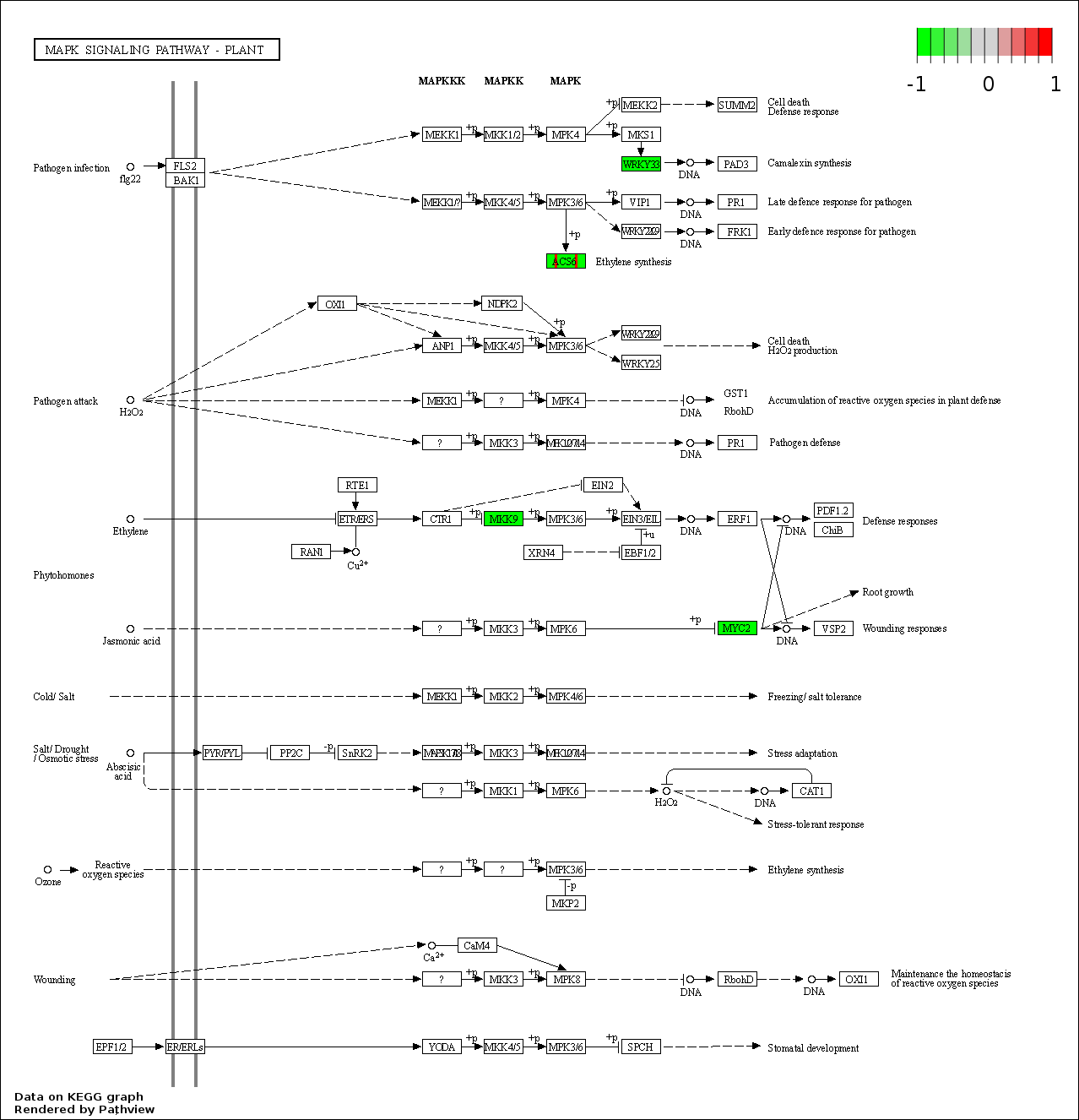


Additional File, Figure 10. Kegg pathway (04016) analysis via Pathview for differentially expressed genes (DEG; absolute log_2_ fold change > 1.5, Benjamini & Hochberg adjusted p-value less than 0.05) in resistant samples (R), compared to susceptible non-diseased samples (S-ND). Color represents expression pattern. Genes without expression information were either not differentially expressed or *Carya illinoinensis* DEGs had no corresponding *Arabidopsis thaliana* ortholog information. Expression pattern for each case sample (R) is represented in each gene box. Reproduction of KEGG pathway granted by Kanehisa Laboratories under open access license.


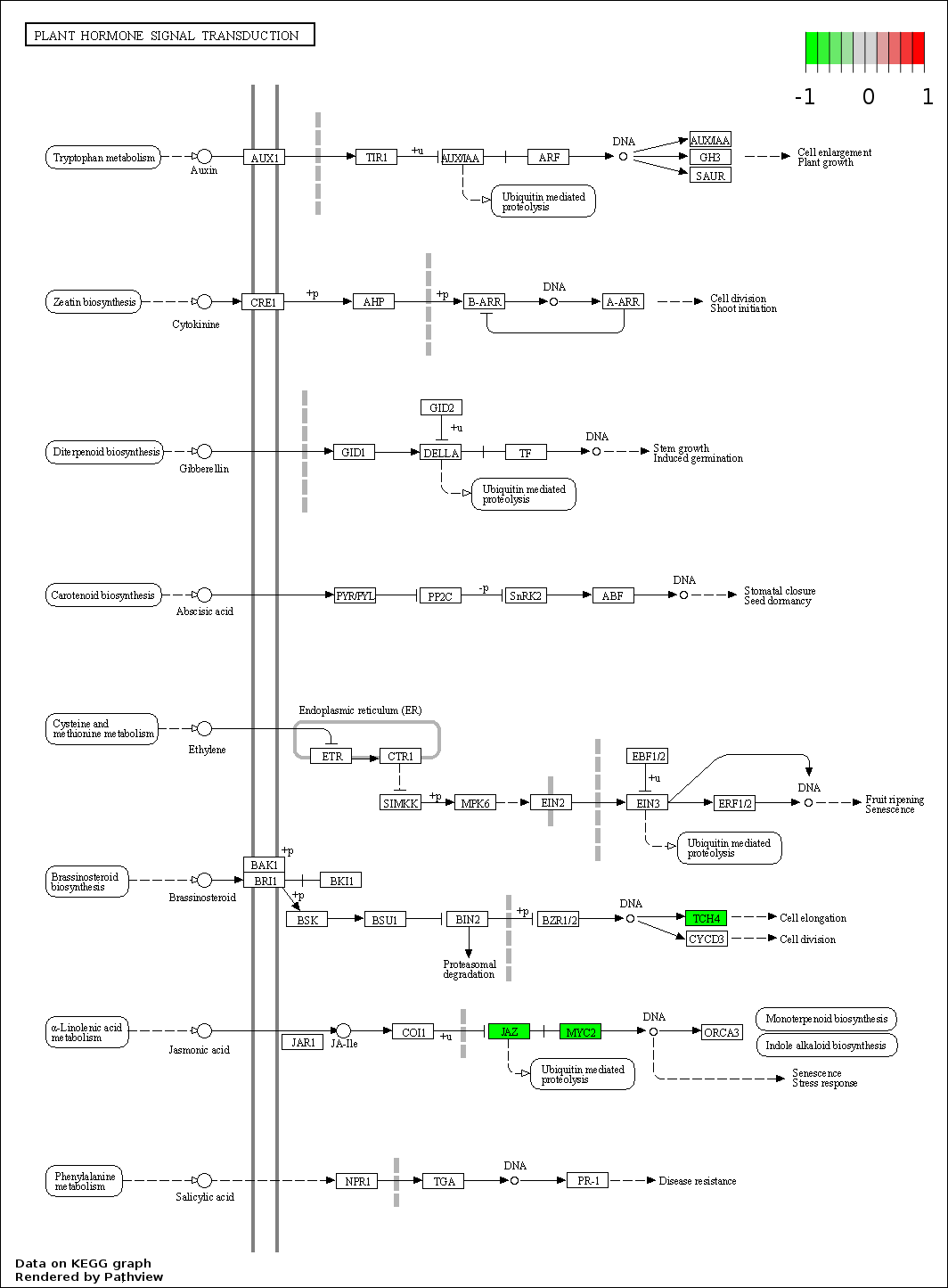


Additional File, Figure 11. Kegg pathway (04075) analysis via Pathview for differentially expressed genes (DEG; absolute log_2_ fold change > 1.5, Benjamini & Hochberg adjusted p-value less than 0.05) in resistant samples (R), compared to susceptible non-diseased samples (S-ND). Color represents expression pattern. Genes without expression information were either not differentially expressed or *Carya illinoinensis* DEGs had no corresponding *Arabidopsis thaliana* ortholog information. Expression pattern for each case sample (R) is represented in each gene box. Reproduction of KEGG pathway granted by Kanehisa Laboratories under open access license.


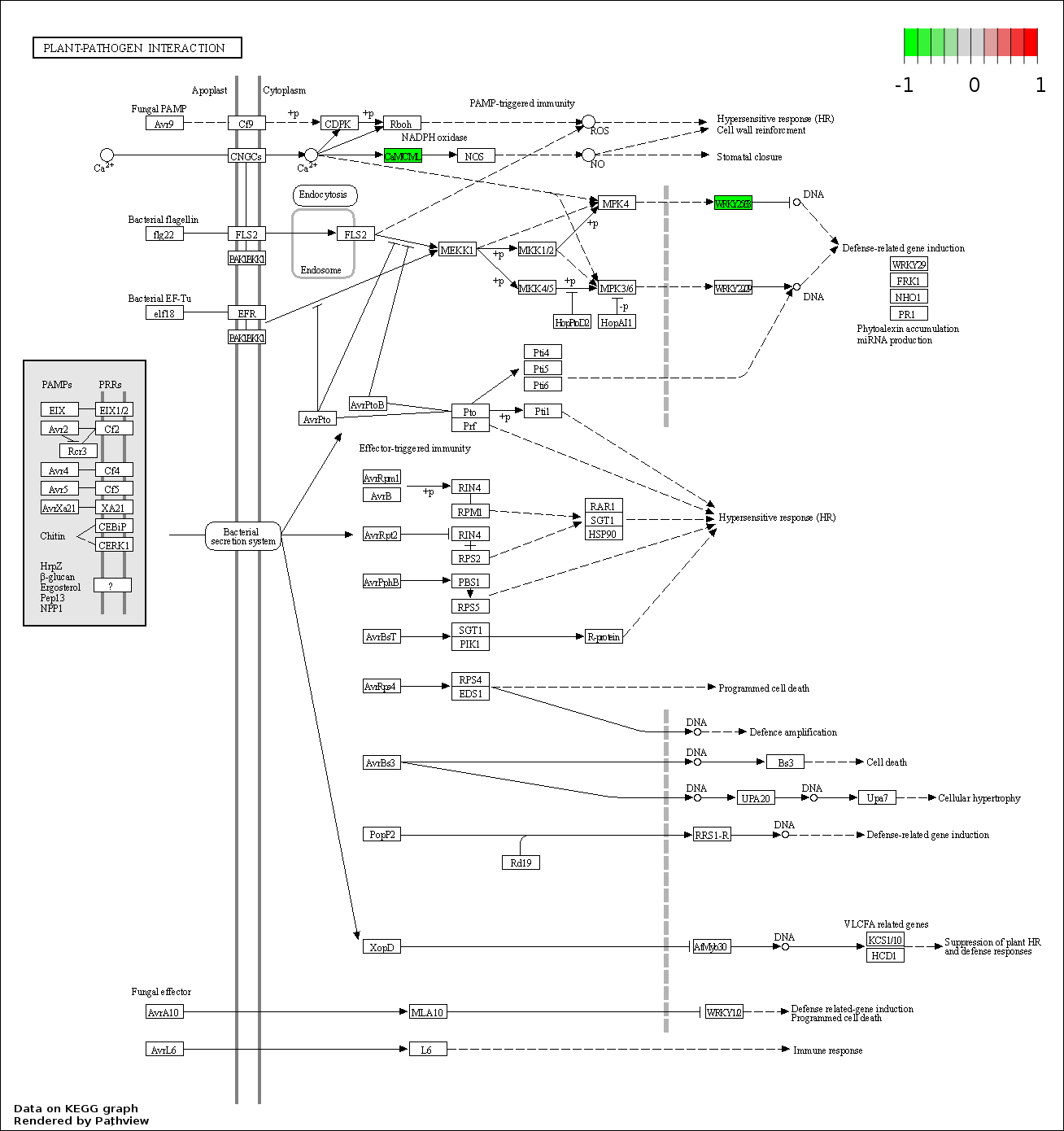


Additional File, Figure 12. Original Kegg pathway (04626) analysis via Pathview for differentially expressed genes (DEG; absolute log_2_ fold change > 1.5, Benjamini & Hochberg adjusted p-value less than 0.05) in resistant samples (R), compared to susceptible non-diseased samples (S-ND). Color represents expression pattern. Genes without expression information were either not differentially expressed or *Carya illinoinensis* DEGs had no corresponding *Arabidopsis thaliana* ortholog information. Expression pattern for each case sample (R) is represented in each gene box. Reproduction of KEGG pathway granted by Kanehisa Laboratories under open access license.


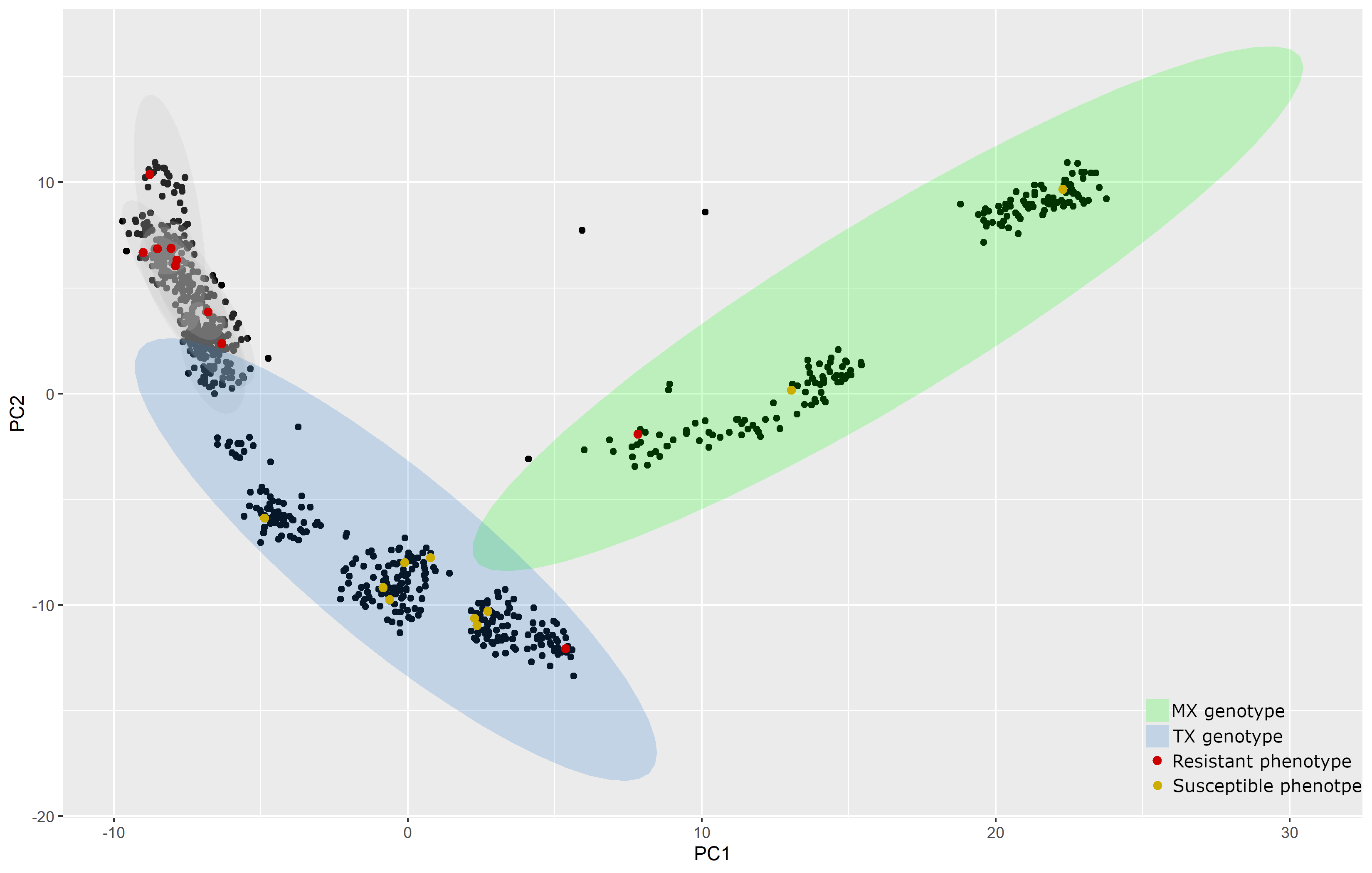


Additional File, Figure 13. Principal component analysis showing the genetic diversity of 835 trees of the pecan provenance collection in Byron, GA using genotype by sequencing (data not shown). Scab susceptible and resistant individuals used in the present study are shown as well as Mexico and Texas genotype clustering. Cultivars developed from northern provenance trees (not Mexico or Texas germplasms) are generally considered to be more resistant to pecan scab.
